# Breast spindle cell tumours: about eight cases

**DOI:** 10.1186/1746-1596-1-13

**Published:** 2006-07-22

**Authors:** Howayda S Abd El All

**Affiliations:** 1 Suez Canal University, Ismailia, Egypt

## Abstract

**Background:**

Breast spindle cell tumours (BSCTs), although rare, represent a heterogeneous group with different treatment modalities. This work was undertaken to evaluate the utility of fine needle aspiration cytology (FNAC), histopathology and immunohistochemistry (IHC) in differentiating BSCTs.

**Methods:**

FNAC of eight breast masses diagnosed cytologically as BSCTs was followed by wide excision biopsy. IHC using a panel of antibodies against vimentin, pan-cytokeratin, s100, desmin, smooth muscle actin, CD34, and CD10 was evaluated to define their nature.

**Results:**

FNAC defined the tumors as benign (n = 4), suspicious (n = 2) and malignant (n = 3), based on the cytopathological criteria of malignancy. Following wide excision biopsy, the tumors were reclassified into benign (n = 5) and malignant (n = 3). In the benign group, the diagnosis was raised histologically and confirmed by IHC for 3 cases (one spindle cell lipoma, one myofibroblastoma and one leiomyoma). For the remaining two cases, the diagnosis was set up after IHC (one fibromatosis and one spindle cell variant of adenomyoepithelioma). In the malignant group, a leiomyosarcoma was diagnosed histologically, while IHC was crucial to set up the diagnosis of one case of spindle cell carcinoma and one malignant myoepithelioma.

**Conclusion:**

FNAC in BSCTs is an insufficient tool and should be followed by wide excision biopsy. The latter technique differentiate benign from malignant BSCTs and is able in 50% of the cases to set up the definite diagnosis. IHC is of value to define the nature of different benign lesions and is mandatory in the malignant ones for optimal treatment. Awareness of the different types of BSCTs prevents unnecessary extensive therapeutic regimes.

## Background

BSCTs although rare, include benign and malignant lesions, necessitating different therapeutic approaches. Within the benign group that may mimics carcinoma clinically and radiologically, and are treated by conservative approach, one should differentiate between spindle cell lipoma (SCL) [1,2] myofiboblastoma (MFB) [3], fibromatosis [4,5], myoepithelioma (ME) [6,7], leiomyoma [8,9], solitary fibrous tumor (SFT) [10], nodular fasciitis [4,11], and benign nerve sheath tumor [12,13]. Noteworthy, although benign in nature, some these tumors such as fibromatosis and SFT have tendency for local recurrence [4,5,11]; therefore the distinction between them is of prognostic significance. In the malignant BSCTs, should be identified, malignant phyllode tumor (PT) [14,15], leiomyosarcoma [16,17] malignant myoepithelioma (MME) [6,7], malignant peripheral nerve sheath tumor [18], spindle cell carcinoma (SCC) [19,20] and metastatic spread from sarcomas.

This study emphasizes the combined role of FNAC and histopathology and the importance of IHC in BSCTs as the treatment modalities are different and crucial for patients' care.

## Materials and methods

### Patients and tissues

The study involved eight female cases complaining of breast masses and referred from surgeons for FNAC. Clinically, all lesions were highly suggestive of malignancy. The initial cytological diagnosis was that of BSCTs. Malignancy was ruled out in four cases due to [1] lack of cytologic atypia, [2] absence of necrosis, and [3] paucity of mitotic figures. Wide excision biopsy enabled to differentiate between benign and malignant BSCTs but was insufficient to reach a final definite diagnosis. Therefore, the precise nomination and the cell of origin or the nature of the lesions was based on IHC.

### Immunohistochemistry

IHC was effectuated on 5 μm thick paraffin embedded tissue sections. The antibodies in the study, their sources, clones, the heat induced epitope antigen retrieval (HIER) and dilutions are illustrated in table 1. HIER was done by heating the slides in microwave (800 watts) for 15 minutes (3 cycles × 5 minutes).

**Table 1:** The antibodies used in the study

Antibodies	Source	Antibody	HIER	Dilution	Staining interpretation
Vimentin	DakoCytomation	Mo V9	2	1:50	Cytoplasmic
Cytokeratin	DakoCytomation	Mo AE1/AE3	1	1: 50	Cytoplasmic
S100	DakoCytomation	Rabbit polyclonal	1	1: 500	Cytoplasmic +/-nuclear
Desmin	DakoCytomation	Mo D33	2	prediluted	Cytoplasmic
SMA	DakoCytomation	Mo 1A4	2	1:50	Cytopmasmic
CD34	Biogenex	Mo QBEnd 10	2	Prediluted	Membranous
CD10	Novocastra	NCL-CD10-270	1	1:50	Membranous

Mo: mouse monoclonal.

1-Tris-EDTA pH9.0.

2-Citrate buffer pH6.0.

In brief, slides were hydrated in descending grades of alcohol followed by distilled water. Endogenous peroxidase activity was quenched by 0.3% hydrogen peroxide for 5 minutes followed by rinsing in distilled water and three times wash in phosphate buffer saline (PBS) Ph 7.4. The antibodies were incubated for 30 minutes at room temperature, and then the slides were rinsed in successive bathes of PBS. The revelation was done by the LSAB-2 detection kit (Dakocytomation) according to the manufacturer's instructions. Finally, diaminobenzidine tetrachloride (DAB) was applied for 5 minutes. Slides were counterstained in Harris haematoxylin (Hx), dehydrated, cleared in xyelene and coverslipped.

The control tissue for all antibodies was the normal breast tissue. In addition, blood vessels inside the tumors were used as an internal control tissue for all the antibodies except cytokeratin. Ommittment of the antibodies were used as negative control for the procedure. The staining interpretation is summarized in table 1.

IHC results were evaluated in semi-quantitative manner as following: 0, negative staining with positive control, -/+ when rare cells not exceeding 10% of the total population are positive, +/- when positivity is between 10 % and 50%, and + when more than 50% of the cells are positive.

## Results

**Figure 1:**
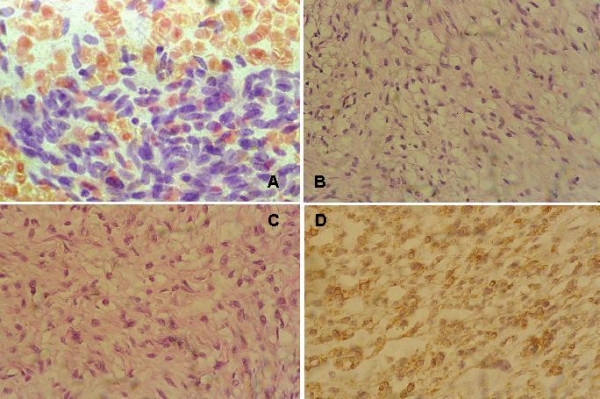
**spindle cell lipoma**. **(a) **Benign cytology BSCT, moderately cellular smear with benign looking spindle cells, Papanicolaou × 400. **(b) **Bland spindle cells intermingled with mature adipocytes and thick collagen bundles, H&E × 400. **(c) **Another field of the same case where the adipocytes are less prominent compared to "b", H&E × 400. **(d) **Adipocytes and spindle cells positive for CD34 IHC, DAB, Hx, × 400. See additional files 1, 2, 3, 4 for higher resolution images.

The age group for the BSCTs ranged from 37–69 years with a median age of 50.37 years. The common denominator on FNAC was the presence of spindle cells without ductal epithelial cells. Lesions diagnosed as benign BSCTs were formed by variable mixtures of spindle, oval and ovoid cells with bland nuclei, inconspicuous nucleoli and low mitotic rate (fig 1a). The presence of discohesive cells with mitotic activity, nuclear atypia and abnormal mitosis in a necrotic background favoured the diagnosis of malignant tumours (Fig 4a, b). In two controversial cases, suspicion of malignancy was raised. The reports were signed out as begnin, suspicious or malignant BSCTs. Cytological evaluation was followed by wide excision biopsy. Grossly, all the lesions were well circumscribed except two. Histologically, the main finding of all the lesions was the presence of spindle cells. Here also, the bland cellular morphology of the spindle cells, mitotic activity not exceeding 2 mitosis/10 high power fields (hpfs) and absence of necrosis, qualify the lesions as benign. Otherwise, the lesion was diagnosed as malignant. Depending on the different proportions of other cellular component, a proposed diagnosis or a differential diagnosis was based. Table 2 summarizes the patients' pathological data and table 3 the IHC profile of the diagnosed cases. Figures 1 through 5 illustrate the cytological, histopathological and IHC findings of some cases.

**Figure 2:**
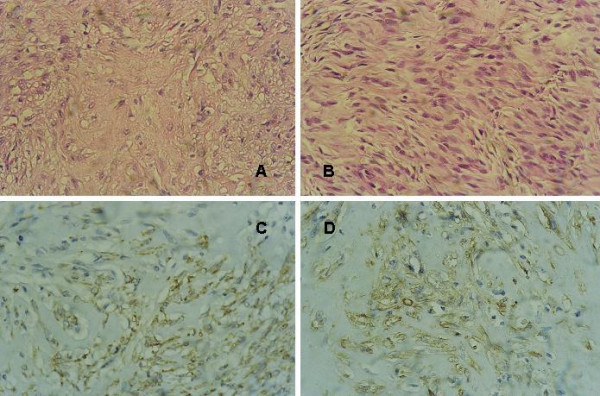
**myofibroblastoma**. **(a) **Most of the tumor is formed of hyalinized collagen bundles surrounded by spindle cells, H&E × 400. **(b) **Cellular area formed of palissading spindle cells, H&E × 400. **(c) **Staining of spindle and oval cells is more pronounced in the lower right part, CD34 IHC, DAB, Hx × 400. **(d) **Focal area of spindle and oval cells positive for SMA, IHC, DAB, Hx ×400. See additional files 5, 6, 7, 8 for higher resolution images.

**Figure 3:**
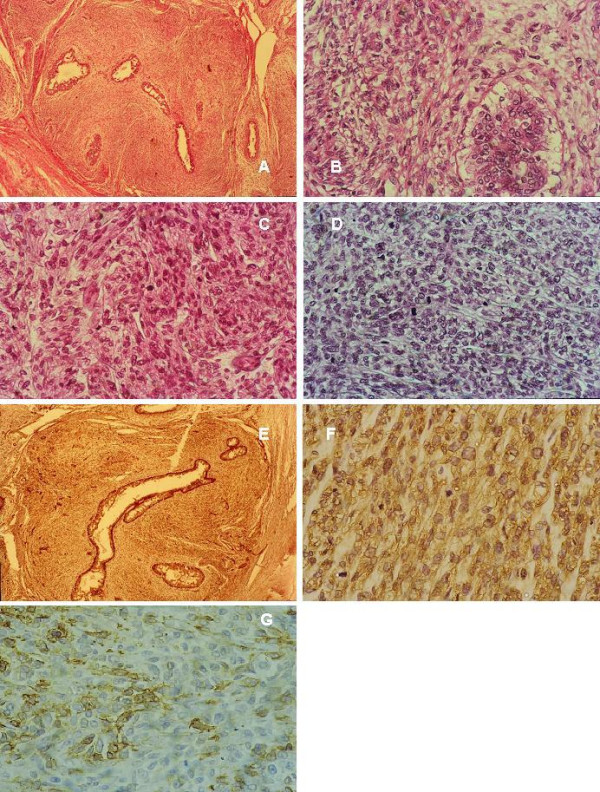
**myoepithelial carcinoma**. **(a) **Spindle cell proliferation surrounding epithelial lumina, H&E × 400. **(b) **Higher power view showing pleomorphic spindle cells with eosinoplilic and clear cytoplasm surrounding an epithelial lined space, H&E × 400. **(c) **Another area showing pleomorphic spindle and ovoid cells with eosinophilic cytoplasm arranged in fascicles; cells have high mitotic activity, H&E × 400. **(d) **Pleomorphic spindle and ovoid cells with clear cytoplasm arranged in wavy fascicles, H&E × 400. **(e) **s100 p IHC staining of the myoepithelial cells around the epithelial lumina and the neoplastic spindle cells × DAB, Hx, ×10. **(f) **Higher magnification of "fig 4e" showing staining of almost all the cells for s100 p IHC, DAB, Hx, × 400. **(g) **CD10 IHC, DAB, Hx, × 400. see additional files 9, 10, 11, 12,13, 14, 15 for higher resolution images.

**Figure 4:**
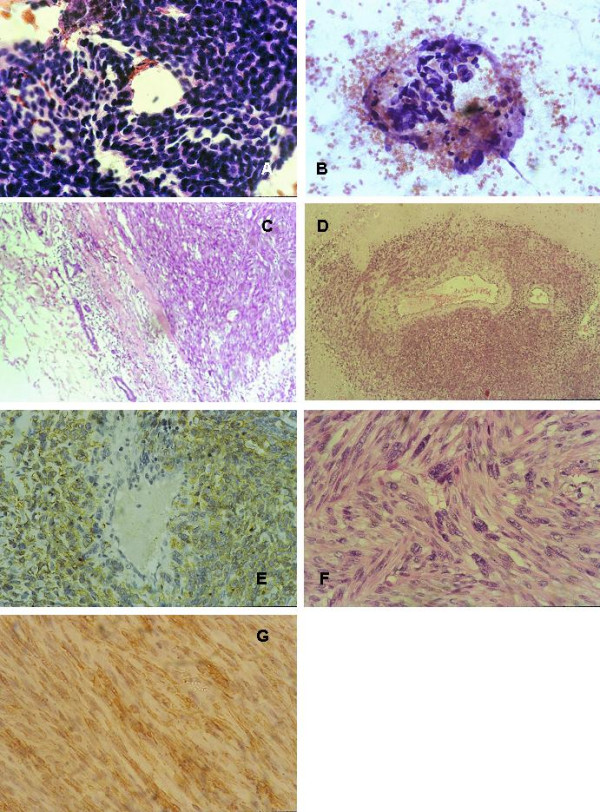
**leiomyosarcoma**. **(a) **Malignant BSCTs cytology, cellular smear with sheets of spindle cells, Papanicolaou staining × 400. **(b) **Another field showing neoplastic ovoid cells, Papanicolaou staining × 400. **(c) **Well circumscribed tumor pushing the normal ductal cells at the periphery, H&E × 10. **(d) **Spindle cells are merging from blood vessels, H&E, × 10. **(e) **Desmin IHC highlighting cells merging from the blood vessels, DAB Hx × 400. **(f) **Intersecting fascicles of pleomorphic malignant spindle cells having cigar shaped blunt ended nuclei, H&E, × 400. **(g) **Desmin IHC, DAB, Hx × 400. See additional files 16, 17, 18, 19,20, 21, 22 for higher resolution images.

**Figure 5:**
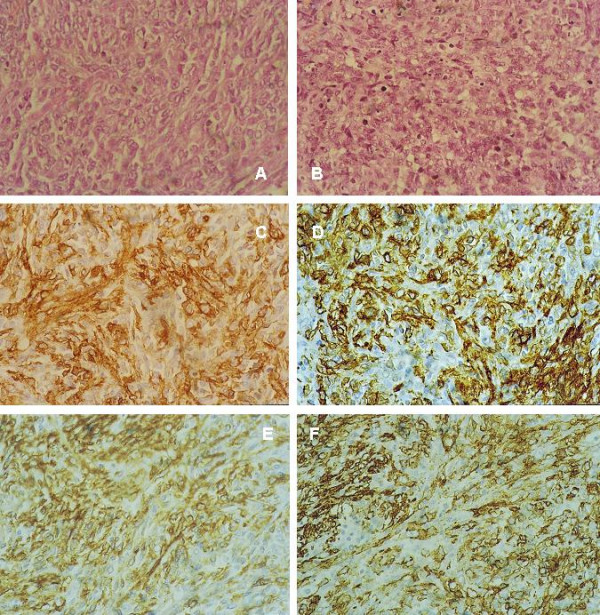
**spindle cell carcinoma**. **(a) **Area with bland morphology compared to "fig 4b", H&E ×400. **(b) **Area with pleomorphic cells, and high mitotic activity, H&E × 400. **(c) **CK IHC, DAB, Hx, × 400. **(d) **CD10 IHC, DAB, Hx, × 400. **(e) **s100p, DAB, Hx, × 400 f- SMA IHC, DAB, Hx, × 400. See additional files 23, 24, 25, 26,27, 28 for higher resolution images.

**Table 2:** Patients' pathological summaries

N°	Age	Cytology	Gross pathology	Histopathology	Provisional diagnosis
1	54	Benign	Well defined, 2 cm	Spindle cells, adipocytes, thick collagen bundles in variable proportions	SCL?
2	55	Suspicious	Well defined, 3 cm	Spindle and oval cells haphazardly arranged in short intersecting fascicles, ribbons of collagen	MFB?
3	46	Benign	Irregular infiltrative, 3.7 cm	Spindle cells forming interlacing bundles	Benign BSCTs?
4	37	Benign	Well defined, 2.5 cm	Cellular neoplasm formed of spindle and oval cells, arranged in storiform pattern or in parallel fascicles	Benign BSCTs?
5	51	Suspicious	Well defined with irregular margins, 3 cm	Highly cellular neoplasm formed of spindle cells surrounding epithelial lined spaces, high mitotic activity 20/10 hpfs	Malignant PT?
6	39	Benign	Well defined, 4 cm	Interlacing bundles of spindle-shaped cells with cigar shaped-nuclei, eosinophilic cytoplasm, rare mitotic figures	leiomyoma
7	52	Malignant	Well defined, 5 cm	Eosinophilic spindle cells arranged in interlacing bundles, marked cellular and nuclear atypia, mitosis 7/10 hpfs, wide areas of necrosis	leiomyosarcoma
8	69	Malignant	Well defined, 4 cm	Highly pleomorphic spindle cells, high mitotic activity 23/10hpfs	Malignant BSCTs?

Hpfs: high power fields.

**Table 3:** IHC profile of the studied cases

	Provisional diagnosis	Vimentin	CK	S100	Desmin	SMA	CD34	CD10	Final diagnosis
1	SCL?	+	-	-	-	-	+	-	SCL
2	MFB?	+	-	-	+	+/-	+	-	MFB
3	Benign BSCTs?	+	-	-	-	+		-	Fibromatosis
4	Benign BSCTs?	-	+	+	-	+/-	-	-/+	ME
5	Malignant PT?	-	+	+	-	-/+		+/-	MME
6	leiomyoma	+	-	-	+	+	-	-	Leiomyoma
7	leiomyosarcoma	+	-	-	+	+	-	-	Leiomyosarcoma
8	Malignant BSCTs?	-/+	+	+	-	+	-	+	SCC

- negative.

-/+ rare cells not exceeding 10% of the total population.

+/-between 10 and 50%.

+ more than 50%.

## Discussion

The diagnosis of the first case (fig 1), SCL was raised on histopathological basis and confirmed after IHC. Histologically, the lesion consisted essentially of spindle cells intermingled with adipocytes and collagen bundles, features described for soft tissue and breast SCL [1,2,21]. However, contrary to the infiltrative nature of the lesion described by Mulvany et al [2], no mammary tissue was seen entrapped in SCL in the present work. In addition, mast cells usually identified in SCL of soft tissue [21] were not found here. IHC confirmed the histological diagnosis and showed reactivity of the spindle cells for vimentin and CD34, finding previously described [2,21].

MFB is a tumor closely related to SCL [22,23] (case 2, fig 2). However, contrary to the begnin cytological nature of SCL, MFB was unobvious based on the presence of nuclear pleomorphism in some spindle cells. This finding is consistent with other studies, cytologically misinterpreting this tumor as malignant soft tissue tumor or PT [24,25]. Histologically, MFB was formed of spindle cells, hyalinized collagen bundles and adipocytes, criteria previously described by in the original study by Wargotz and colleagues[3]. The diagnosis of this case was based on IHC positivity for vimentin, CD34, desmin and focal positivity for SMA, a finding described in the literature [26]. While all the literature agrees on the positivity of MFB for vimentin and CD34, controversies regarding the positivity for desmin and SMA are reported [3,27].

This tumor, of myofibroblastic origin, is capable of diverse lines of differentiation [28]; and based on the proportion of the spindle cells, collagenous stroma and cellular pleomorphism, is subclassified as classic, collagenized, epithelioid, cellular, lipomatous and variants resembling SFT [22,27,29]. Therefore this case was signed out as collagenized MFB. It is crucial to recognize this tumor and to distinguish it from its malignant counterpart, myofibrosarcoma. The latter, characterized by marked cellular pleomorphism, infiltrating margins and high mitotic rate, necessitate more aggressive extensive and radical surgery [30].

At a molecular level, MFB shares cytogenetic abnormalities with SCL [23], leading to the hypothesis of a dual, myofibroblastic and lipomatous, differentiation from a common pluripotential mesenchymal precursor cell, represented by the vimentin+/CD34+ fibroblast of the mammary stroma as suggested by Magro and co-workers [28]. In latter studies, the same investigators postulated that the "vimentin+/CD34+ cell" is the precursor cell of all benign spindle cell neoplasm [31,32]. This hypothesis provides explanation for the phenotypic heterogeneity of these neoplasm and their variable IHC profiles taking into consideration the well-known inherent plasticity of the "vimentin+/CD34+ cell" to differentiate toward several mesenchymal lines.

Breast fibromatosis (case 3) is a lesion that clinically and radiologically suggests breast cancer [33,34]. Fibromatosis is an infiltrative fibroblastic and myofibroblastic proliferation with significant risk for local recurrence, without metastatic potential. Specific histological features, such as size, cellularity, atypia, and mitotic figures, are not helpful in predicting recurrence [5]. The diagnosis of fibromatosis in this work was based on positivity of the spindle cells for vimentin and SMA, a finding described in other studies [35,36].

ME tumors are defined as lesions arising from or composed of a dominant to pure population of myoepithelial cells (ME cells) in the WHO classification 2003 [7]. ME cells are immunoreactive for SMA, CD10, s100, CK and high molecular weight CK [7,37,38]. ME tumors are divided into myoepitheliosis, adenomyoepithelial adenosis, ME and MME. A case of spindle cell ME (case 4) has been diagnosed by IHC. Cells were strongly reactive for CK, s100, SMA and focally positive for CD10. Variable levels of expression for CD10 have been reported depending on the type of ME tumors [39]. It has been postulated that CD10 expression in combination with SMA is more informative on ME cells' nature [40]. A second tumor encountered here of ME origin is MEC (case 5, fig 3). Histologically, it mimicked malignant PT, as it was composed of malignant spindle cells surrounding luminal epithelia. However, based on the negativity of the spindle cells for CD34 and their positivity for CK, s100, SMA and CD10 [37,38], the diagnosis of malignant PT have been excluded. This diagnosis is important in view of the aggressive behaviour of MME compared to malignant PT [41,42].

Two smooth muscle tumors had been encountered in this study, leiomyoma and leiomyosarcoma. These tumors, since their initial description by Strong [8], are rare breast neoplasm. Two types of breast leiomyoma are identified, superficial and vascular leiomyoma. Superficial leiomyomas, are located in the skin and subcutaneous tissues and involve the nipple or areolar region, while vascular leiomyomas are located deep within the breast parenchyma and are less common than the superficial counterpart. The true histogenesis of breast smooth muscle tumors remains unknown. Cells may be considered as hamartomatous proliferation of smooth muscle surrounding blood vessels, embryonal migration of smooth-muscle cells from the nipple, differentiation from multipotent mesenchymal cells in breast tissue or derivation from myoepithelial cells of breast ducts with frank differentiation to smooth muscle [9,43]. The leiomyoma diagnosed in the present work (case 6) was a deep one arising from smooth muscles around blood vessels. Histologically, it demonstrated interlacing bundles of spindle cells with blunt-ended cigar shaped nuclei, and eosinophilic cytoplasm. There was no nuclear atypia, no hypercellularity. The mitotic rate was low and necrosis was absent. However, the importance resides in differentiating leiomyoma from leiomyosarcoma. The latter diagnosis has been made for case 7 (fig 4). On cytological basis, it was difficult to nominate the lesion which was designed as malignant BSCT. The same difficulty has been reported in other studies, as the tumor was misdiagnosed as poorly differentiated ductal carcinoma or as malignant PT [16,17]. By the contrary, histologically, it fulfilled the criteria for diagnosing leiomyosarcoma. In addition, cells were centred on blood vessels, a feature identified for the leiomyoma case. On IHC bases, both leiomyoma and leiomyosarcoma were positive for both SMA and desmin (fig 5).

SCC of the breast, account for less than 1% of invasive carcinoma [44]. It represents a variant of metaplastic carcinoma (MSC), and includes a wide spectrum of lesions with histomorphologic and nuclear features ranging from overtly malignant to mildly atypical. The case presented in this study (case 8, fig 5) was suspicious for malignancy on FNAC, based on the presence of some atypical nuclei in the spindle cells and scattered mitotic activity. This corresponded histologically to bland areas with mildly pleomorphic cells and low mitotic activity, while most of the tumor was formed of frankly malignant cells with a mitotic activity up to 23 mitosis/10 hpfs. Vimentin, CK, SMA s100 and CD10 were positive on the spindle cells while desmin and CD34 were negative. The expression of SMA, s100 and CD10 on SCC favour the current concept of its myoepithelial origin [15,19,45,46]. Controversies in the literature exist concerning the prognosis being better or worse compared to ordinary breast duct carcinoma [19,20,47-49].

## Conclusion

In conclusion, although BSCTs are infrequent, awareness of this category is essential for patient categorization and optimal therapy. The combination of conventional H&E and IHC using a small panel of antibodies is fundamental. The role of cytology is less obvious in this category.

## Abbreviations

BSCTs: breast spindle cell tumors, SCL: spindle cell lipoma, MFB: myofibroblastoma, ME: myoepithelioma, MME: malignant myoepithelioma, SCC: spindle cell carcinoma, SFT: solitary fibrous tumor, PT: phyllode tumor, CK: cytokeratin, SMA: smooth muscle actin, hpfs: high power fields, Hx: hematoxylin

## Competing interests

The author(s) declare that they have no competing interests.

## Supplementary Material

Additional File 1 High resolution image of figure 1a Click here for file

Additional File 2 High resolution image of figure 1b Click here for file

Additional File 3 High resolution image of figure 1c Click here for file

Additional File 4 High resolution image of figure 1d Click here for file

Additional File 5 High resolution image of figure 2a Click here for file

Additional File 6 High resolution image of figure 2b Click here for file

Additional File 7 High resolution image of figure 2c Click here for file

Additional File 8 High resolution image of figure 2d Click here for file

Additional File 9 High resolution image of figure 3a Click here for file

Additional File 10 High resolution image of figure 3b Click here for file

Additional File 11 High resolution image of figure 3c Click here for file

Additional File 12 High resolution image of figure 3d Click here for file

Additional File 13 High resolution image of figure 3e Click here for file

Additional File 14 High resolution image of figure 3f Click here for file

Additional File 15 High resolution image of figure 3g Click here for file

Additional File 16 High resolution image of figure 4a Click here for file

Additional File 17 High resolution image of figure 4b Click here for file

Additional File 18 High resolution image of figure 4c Click here for file

Additional File 19 High resolution image of figure 4d Click here for file

Additional File 20 High resolution image of figure 4e Click here for file

Additional File 21 High resolution image of figure 4f Click here for file

Additional File 22 High resolution image of figure 4g Click here for file

Additional File 23 High resolution image of figure 5a Click here for file

Additional File 24 High resolution image of figure 5b Click here for file

Additional File 25 High resolution image of figure 5c Click here for file

Additional File 26 High resolution image of figure 5d Click here for file

Additional File 27 High resolution image of figure 5e Click here for file

Additional File 28 High resolution image of figure 5f Click here for file
