# Supplementary figures and images for: Breast spindle cell tumours: about eight cases
Source: Diagn Pathol. 2006 Jul 22;1:13. doi: 10.1186/1746-1596-1-13 (PMC1557590; doi:10.1186/1746-1596-1-13)

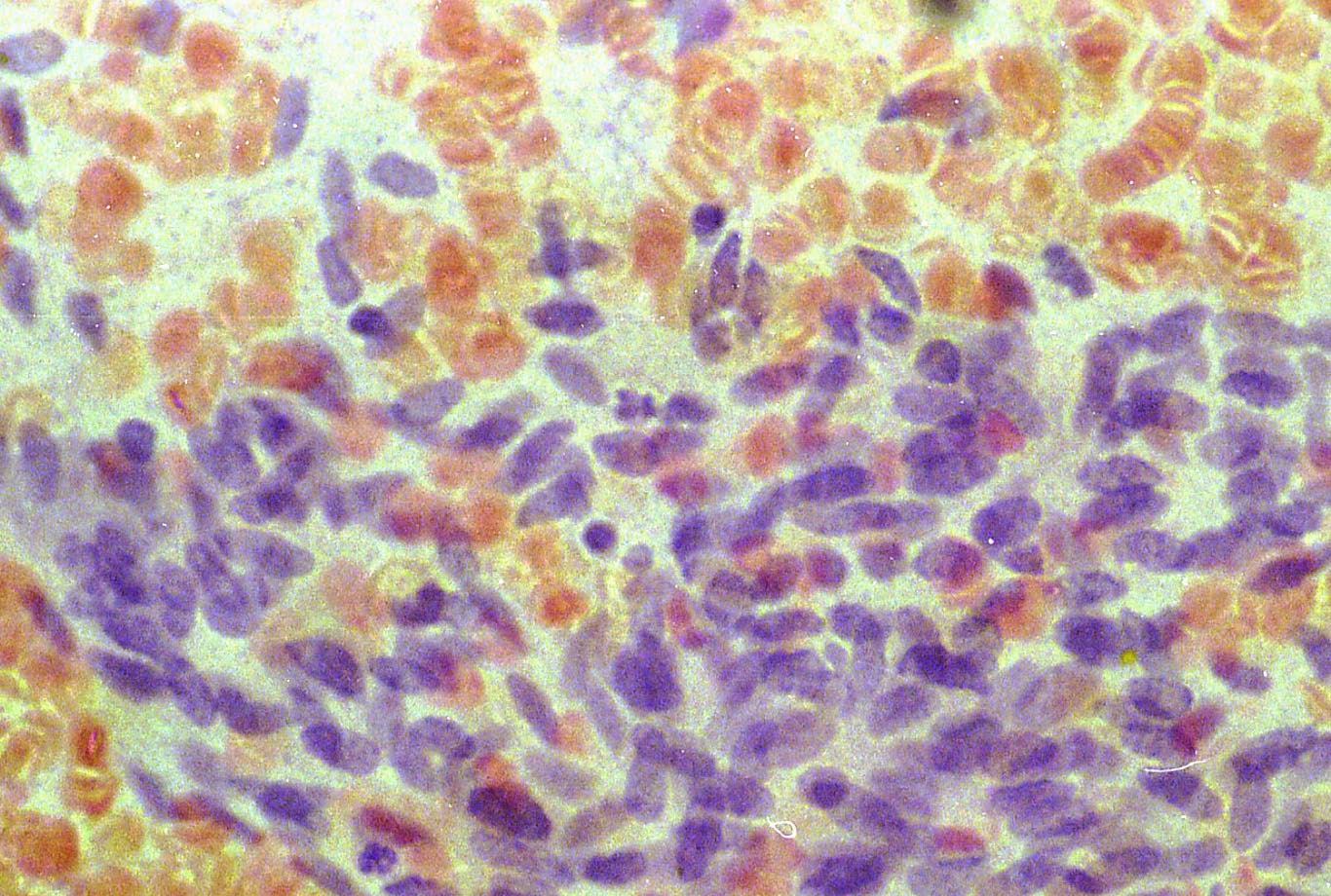

Supplement: Additional File 1 — High resolution image of figure 1a [file 1746-1596-1-13-S1.jpeg]

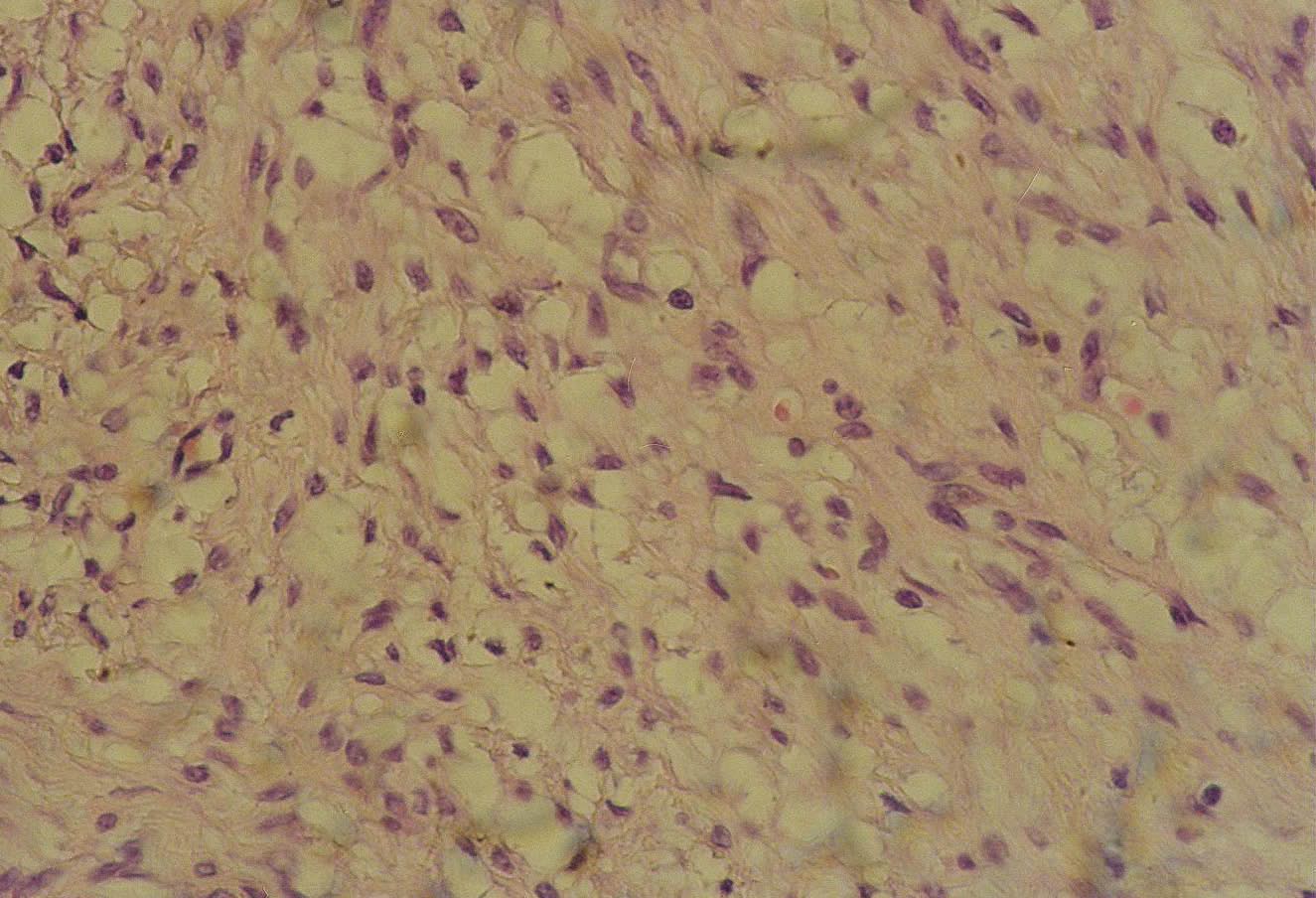

Supplement: Additional File 2 — High resolution image of figure 1b [file 1746-1596-1-13-S2.jpeg]

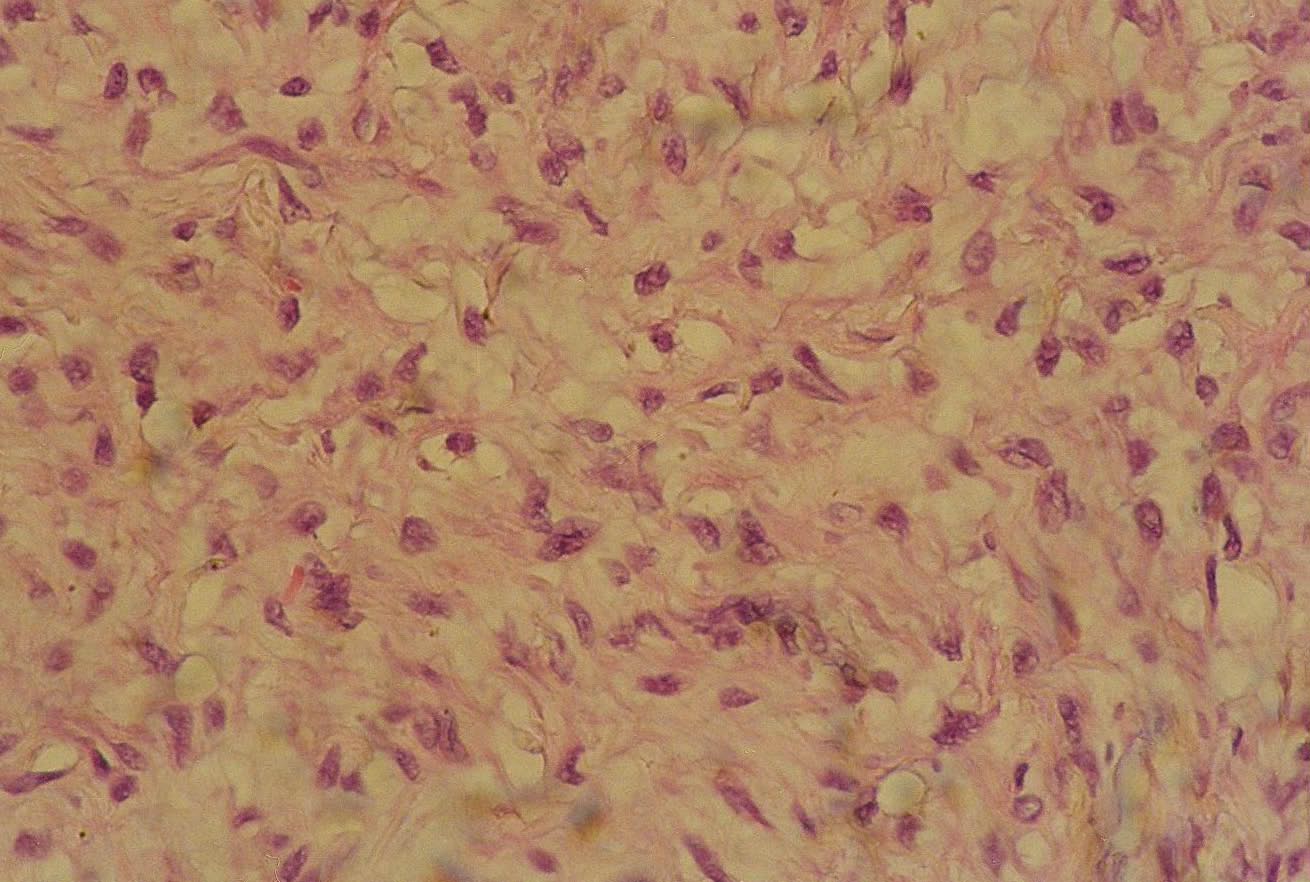

Supplement: Additional File 3 — High resolution image of figure 1c [file 1746-1596-1-13-S3.jpeg]

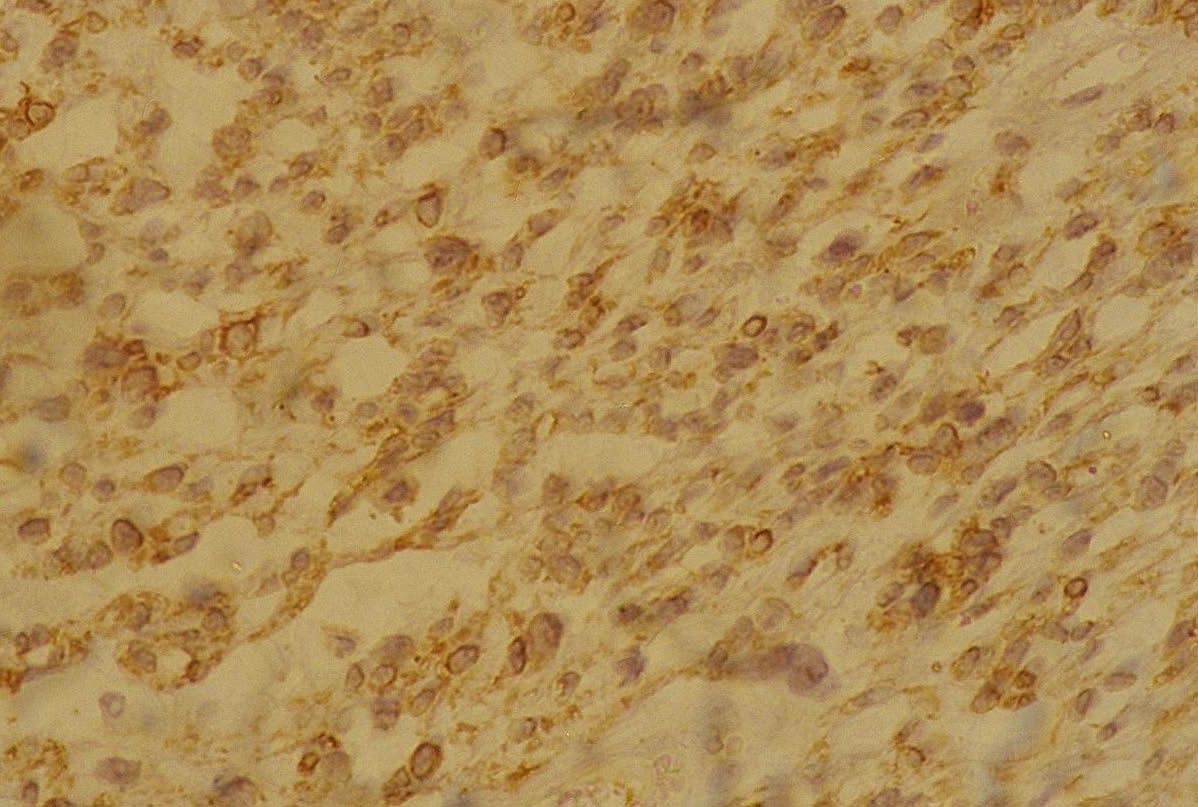

Supplement: Additional File 4 — High resolution image of figure 1d [file 1746-1596-1-13-S4.jpeg]

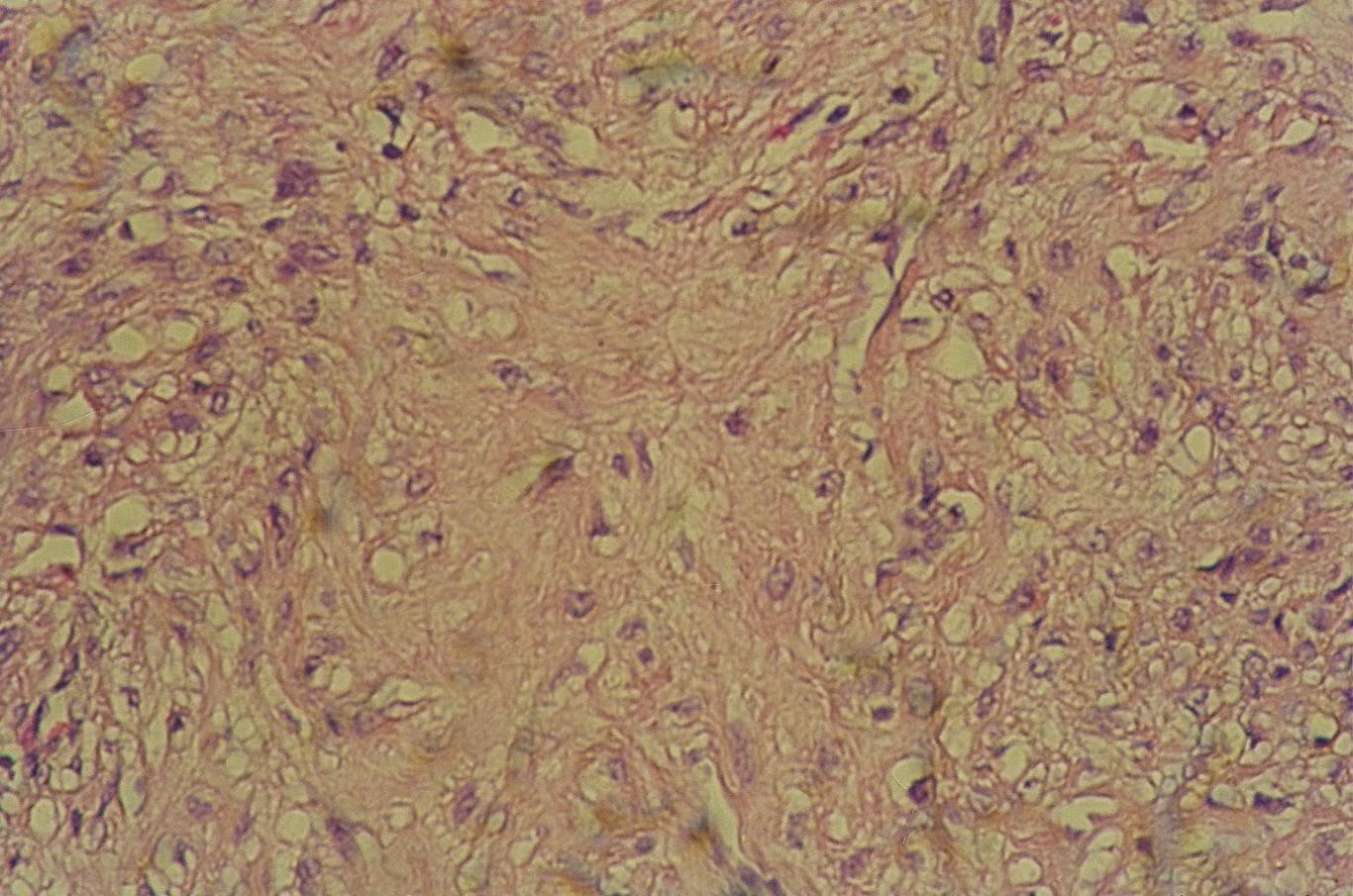

Supplement: Additional File 5 — High resolution image of figure 2a [file 1746-1596-1-13-S5.jpeg]

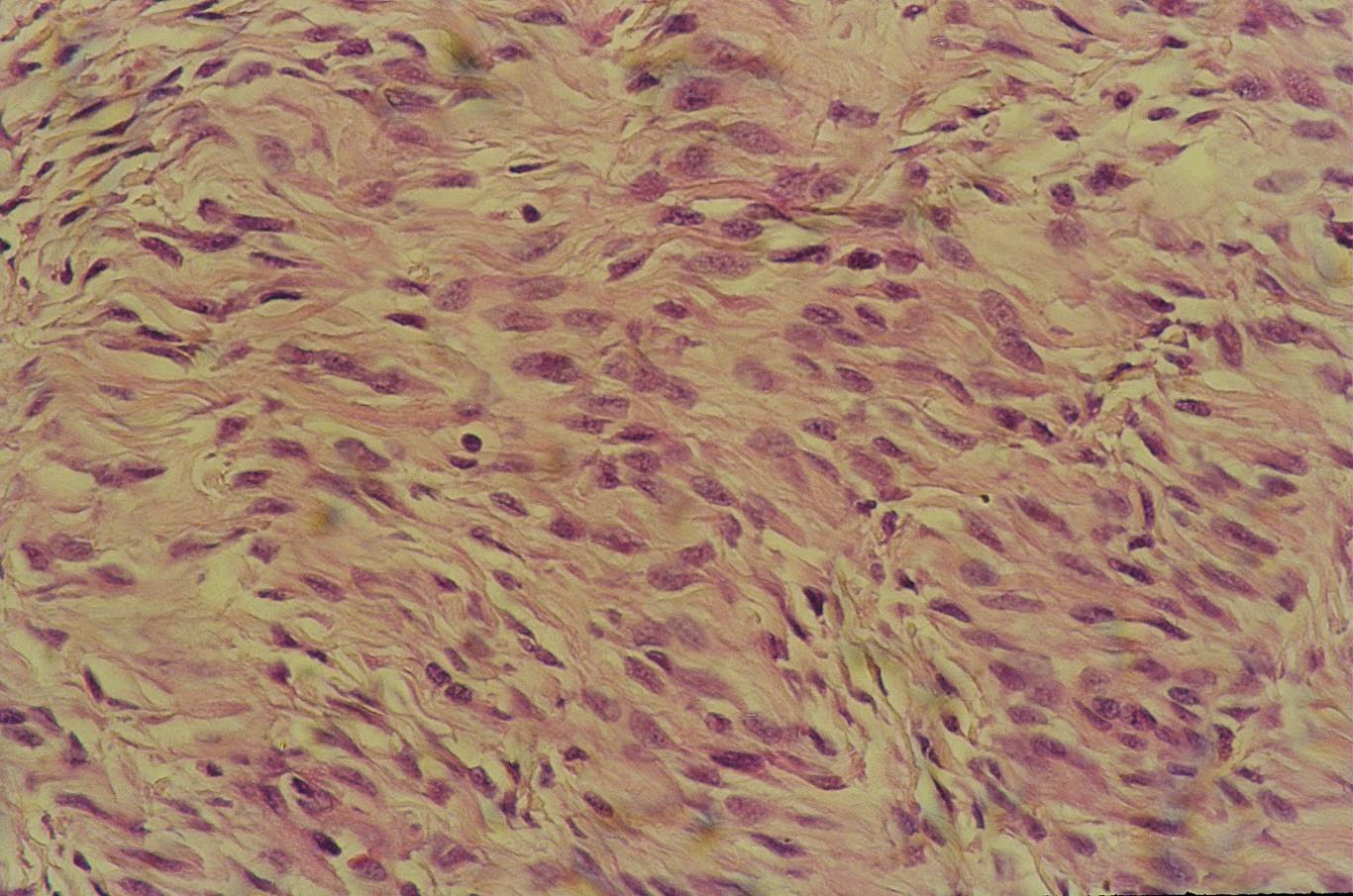

Supplement: Additional File 6 — High resolution image of figure 2b [file 1746-1596-1-13-S6.jpeg]

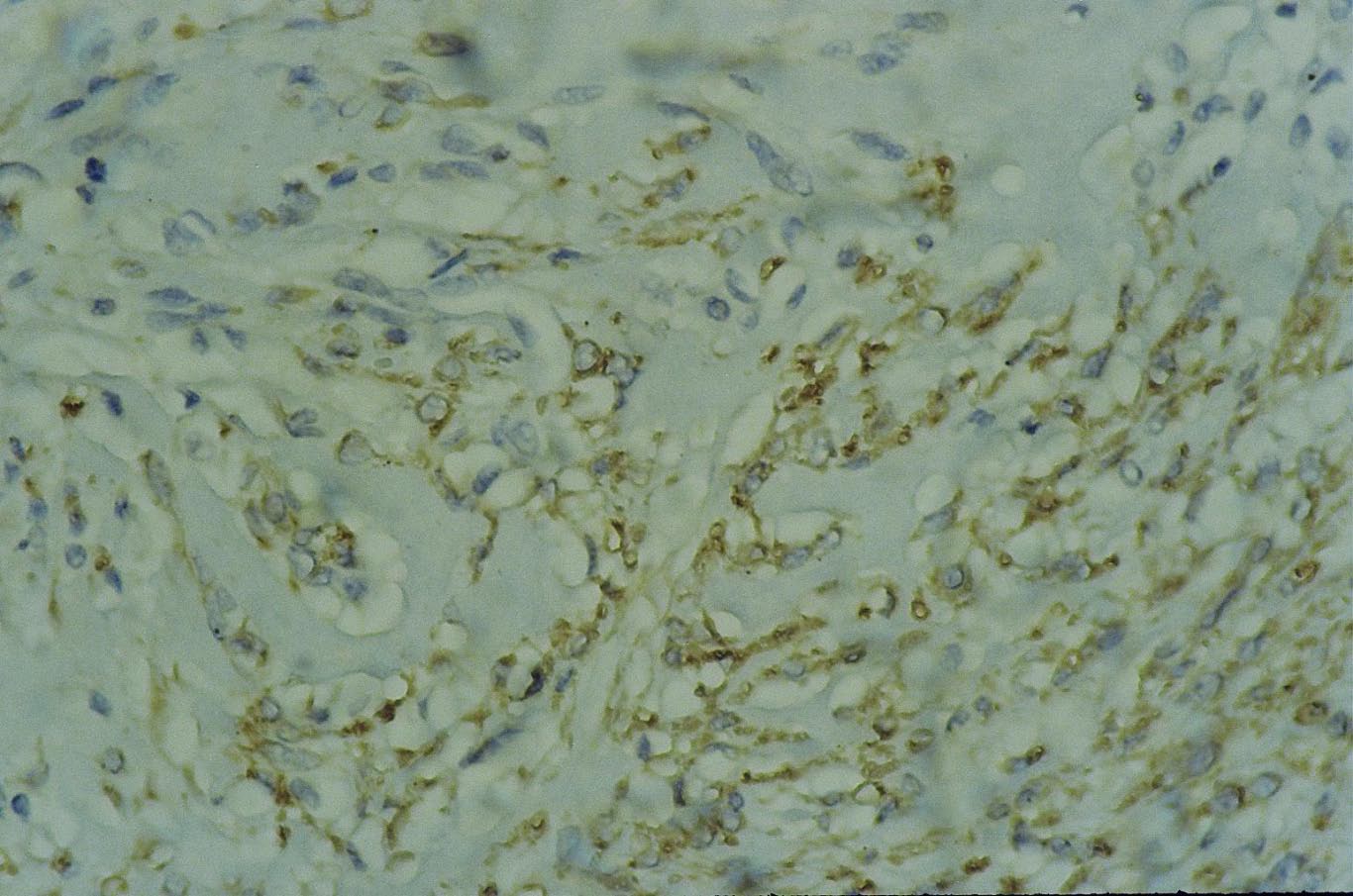

Supplement: Additional File 7 — High resolution image of figure 2c [file 1746-1596-1-13-S7.jpeg]

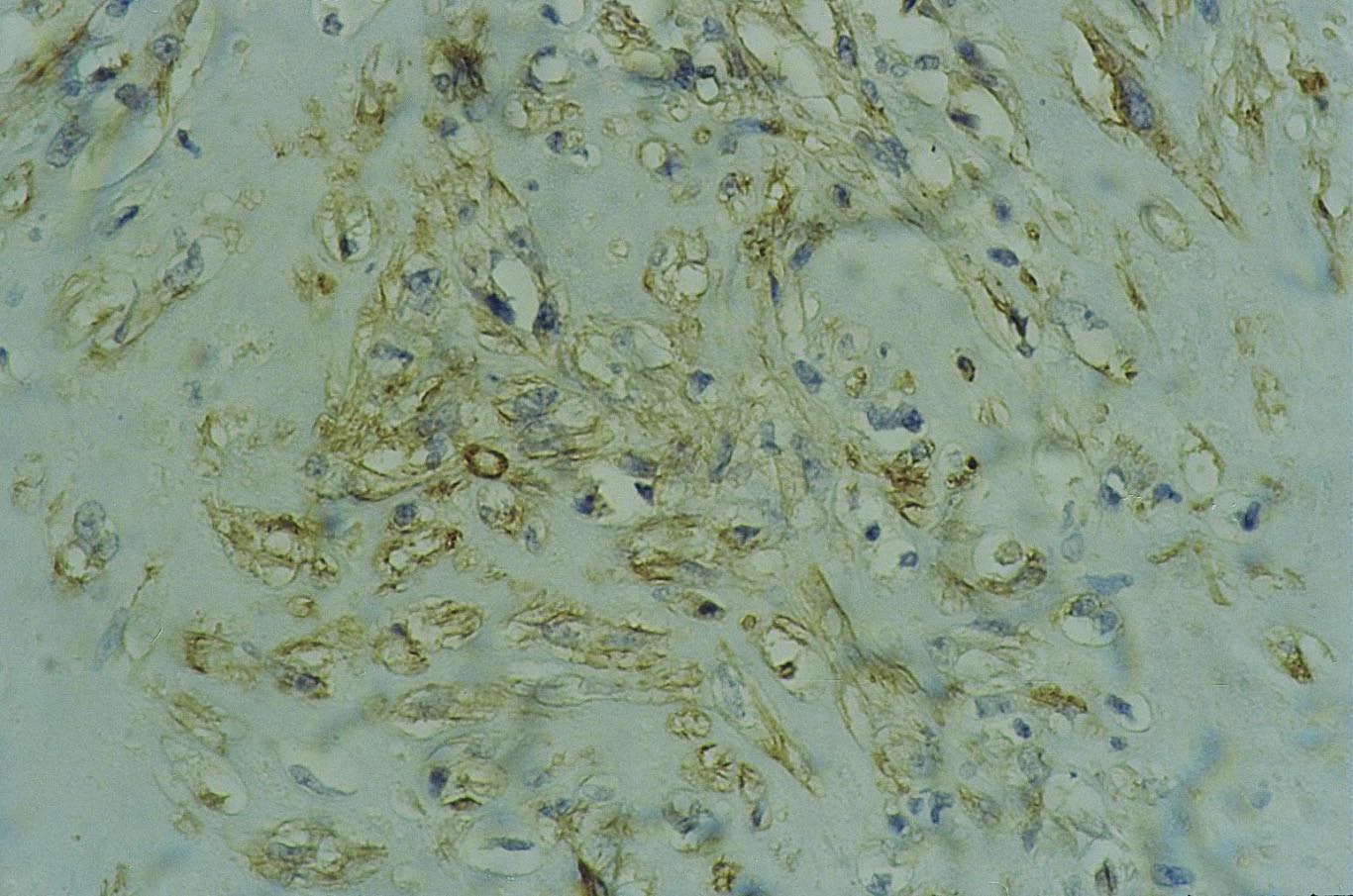

Supplement: Additional File 8 — High resolution image of figure 2d [file 1746-1596-1-13-S8.jpeg]

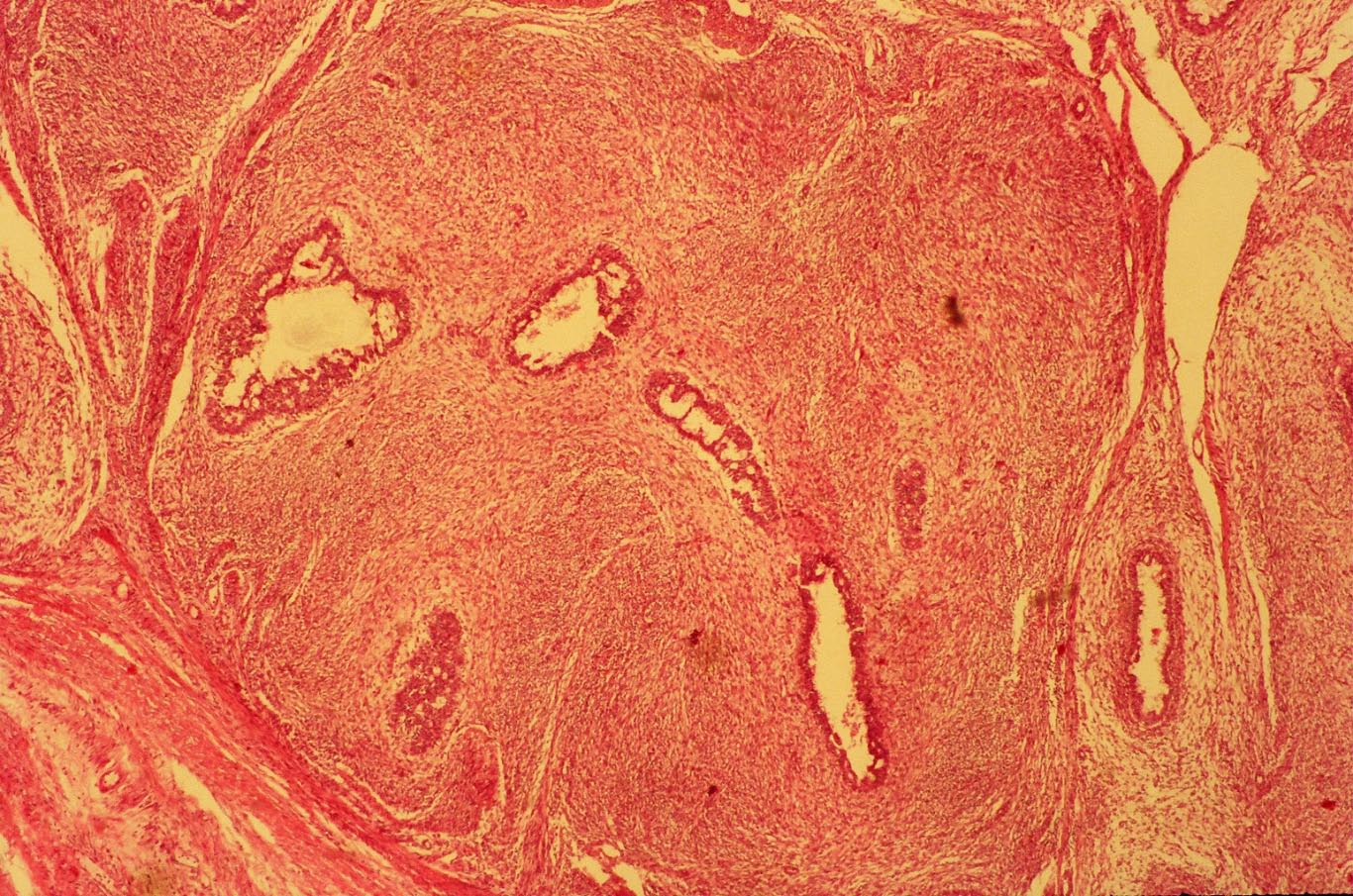

Supplement: Additional File 9 — High resolution image of figure 3a [file 1746-1596-1-13-S9.jpeg]

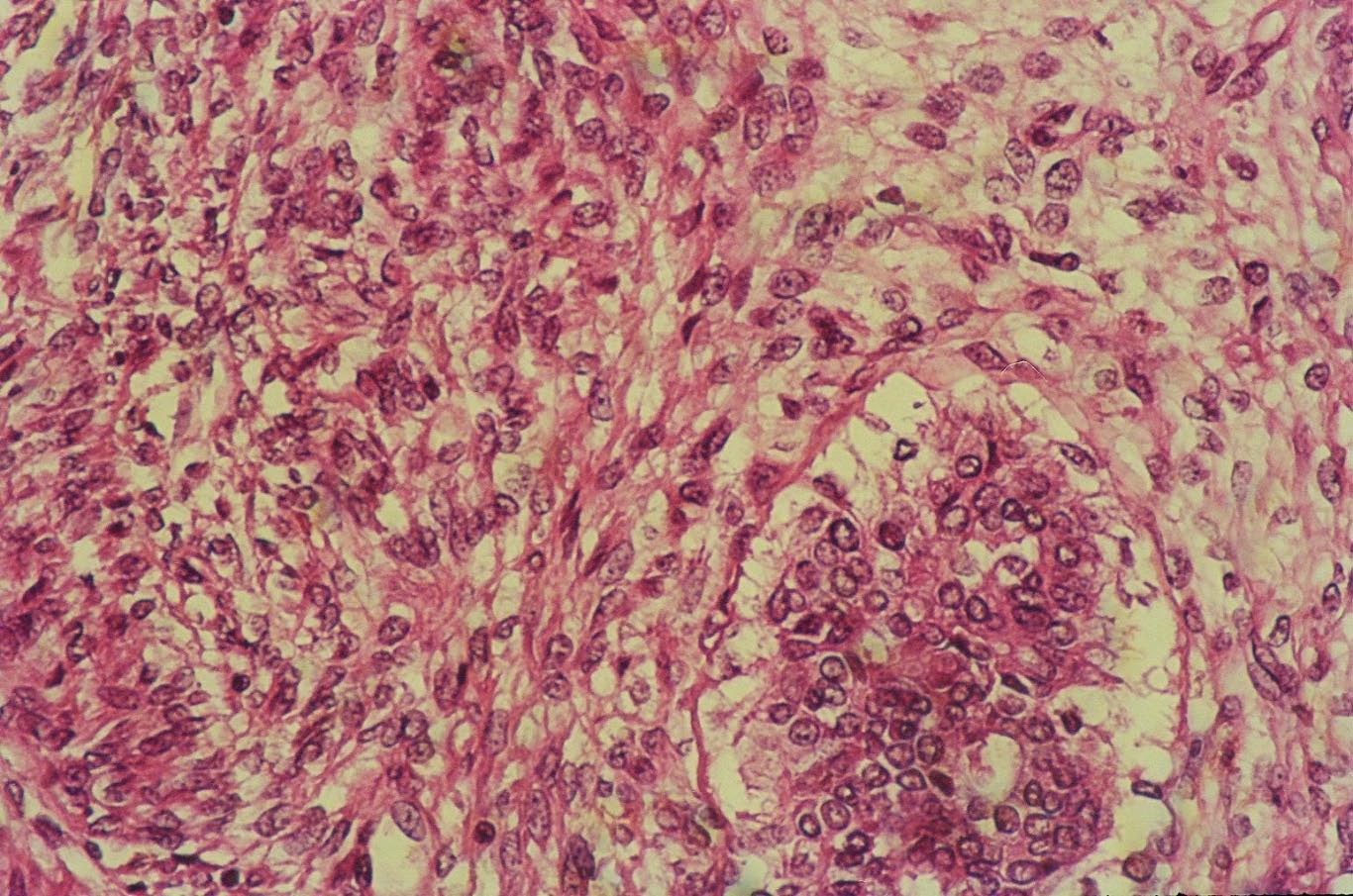

Supplement: Additional File 10 — High resolution image of figure 3b [file 1746-1596-1-13-S10.jpeg]

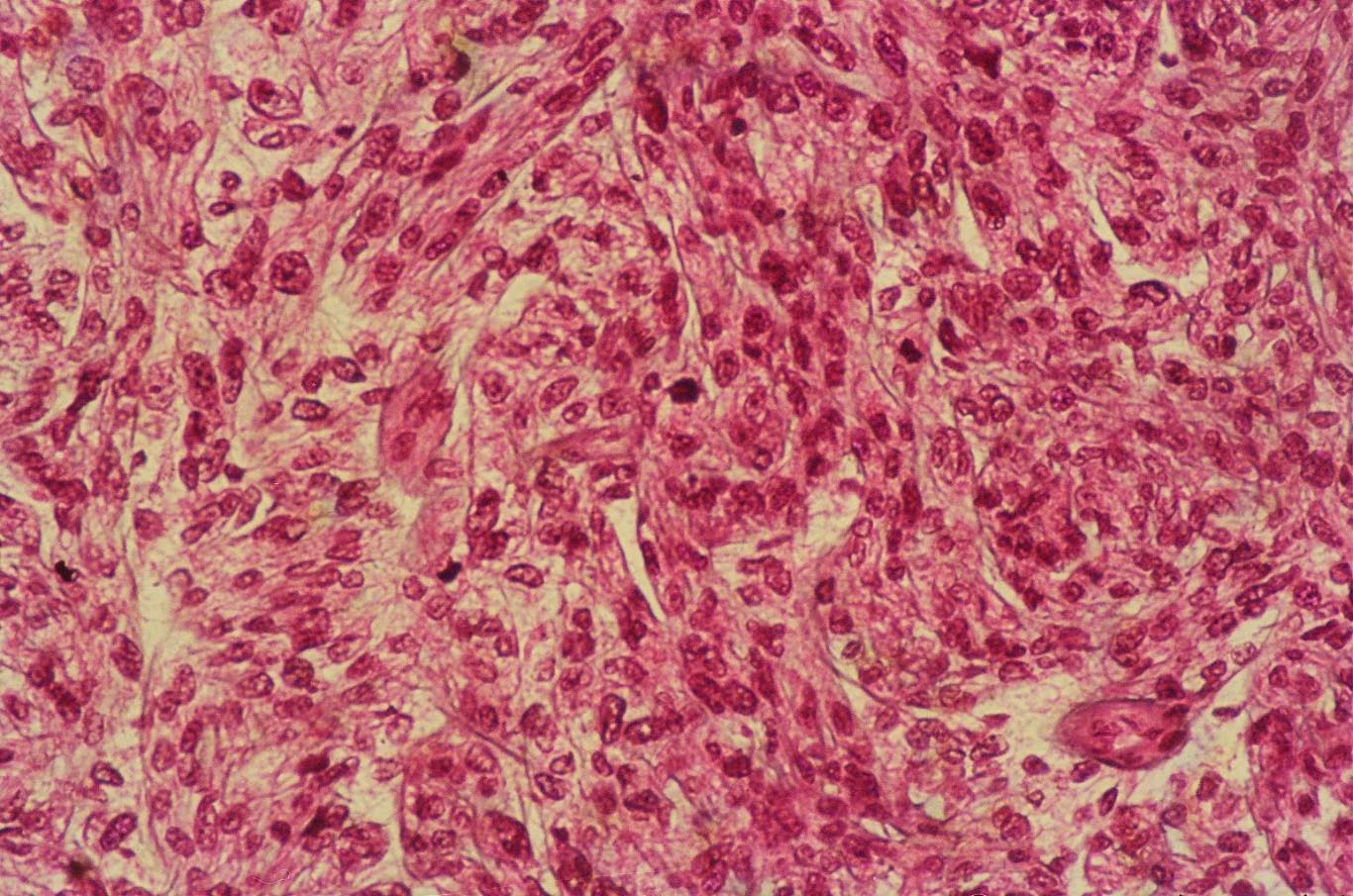

Supplement: Additional File 11 — High resolution image of figure 3c [file 1746-1596-1-13-S11.jpeg]

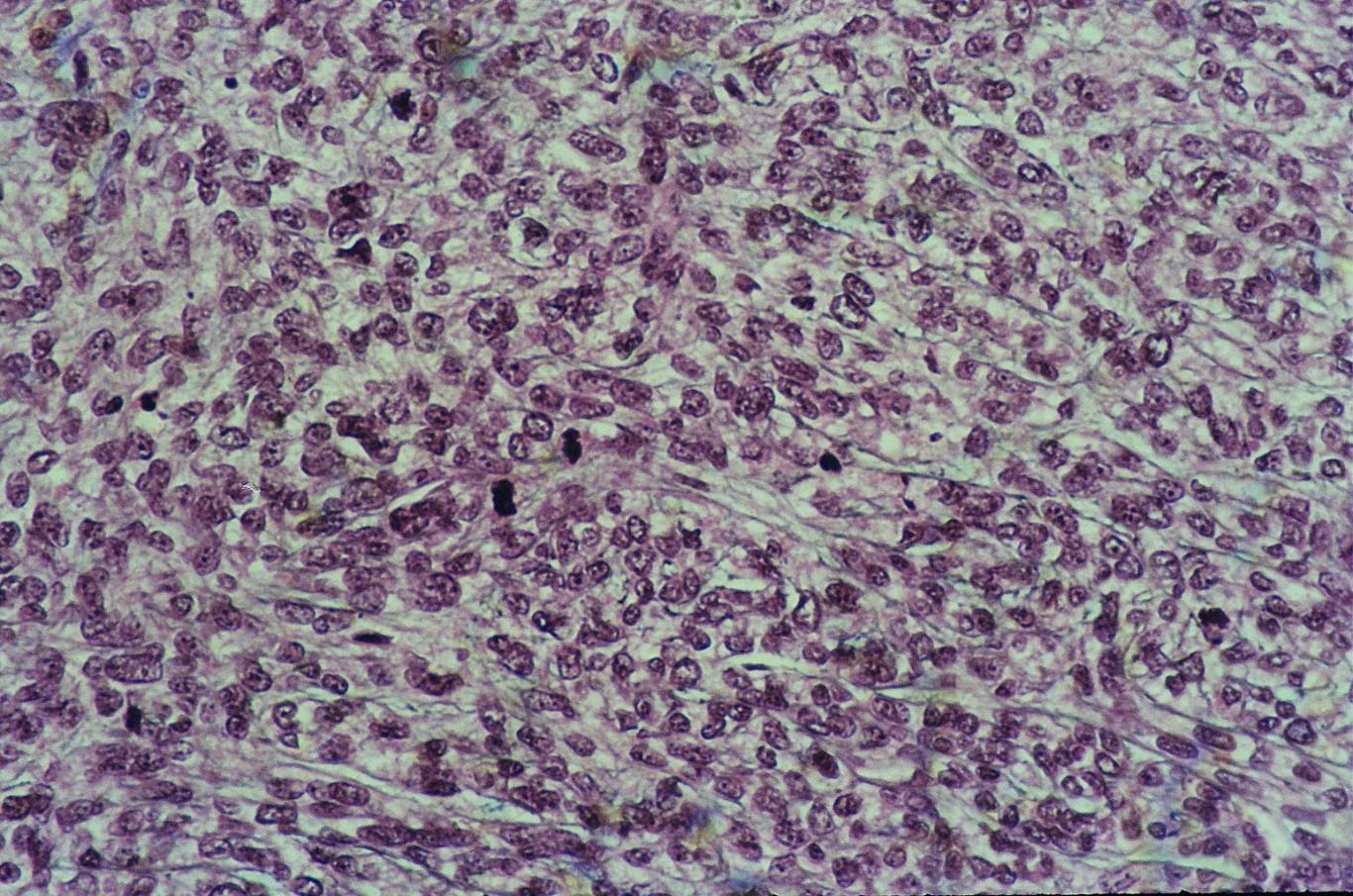

Supplement: Additional File 12 — High resolution image of figure 3d [file 1746-1596-1-13-S12.jpeg]

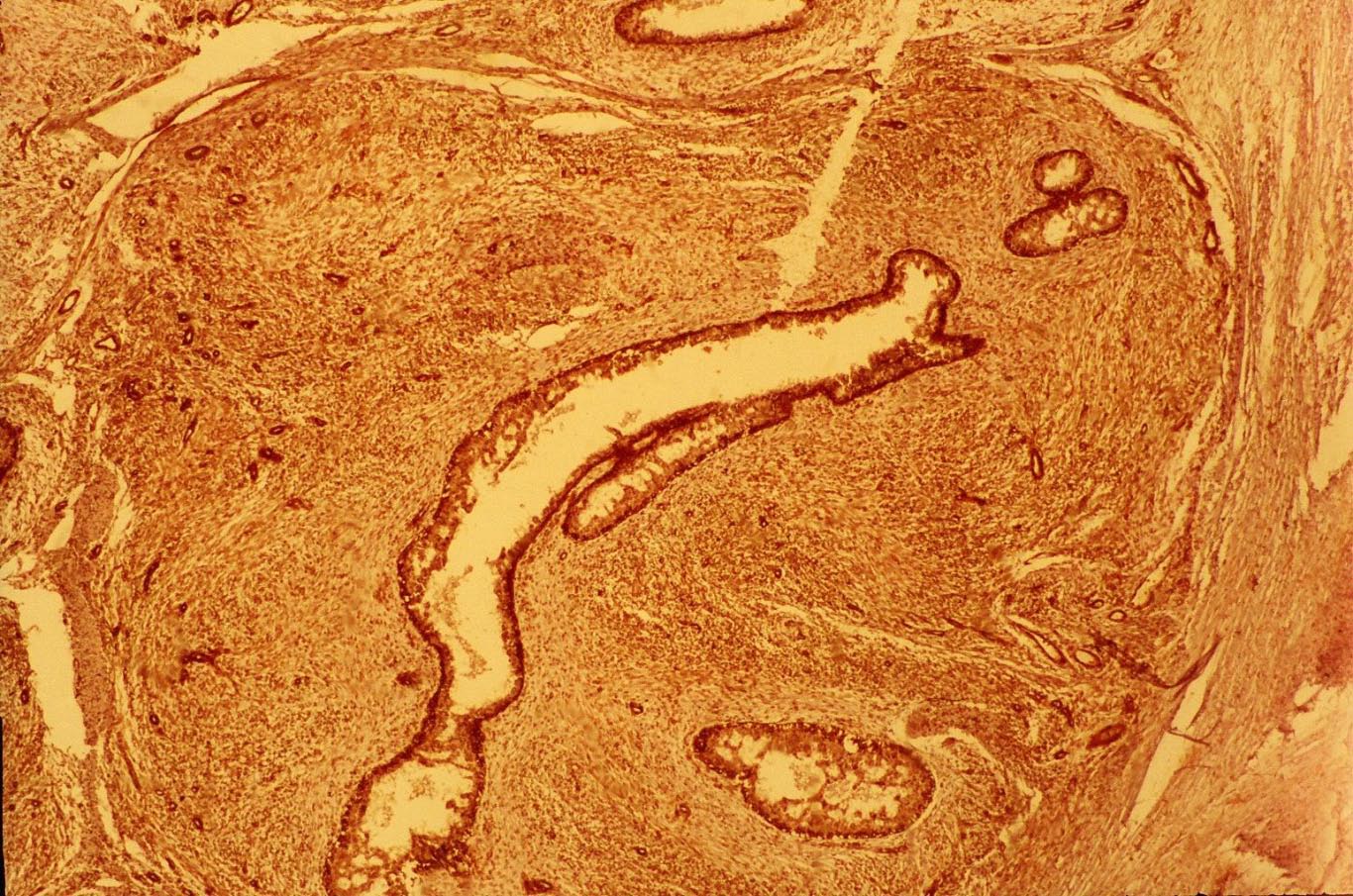

Supplement: Additional File 13 — High resolution image of figure 3e [file 1746-1596-1-13-S13.jpeg]

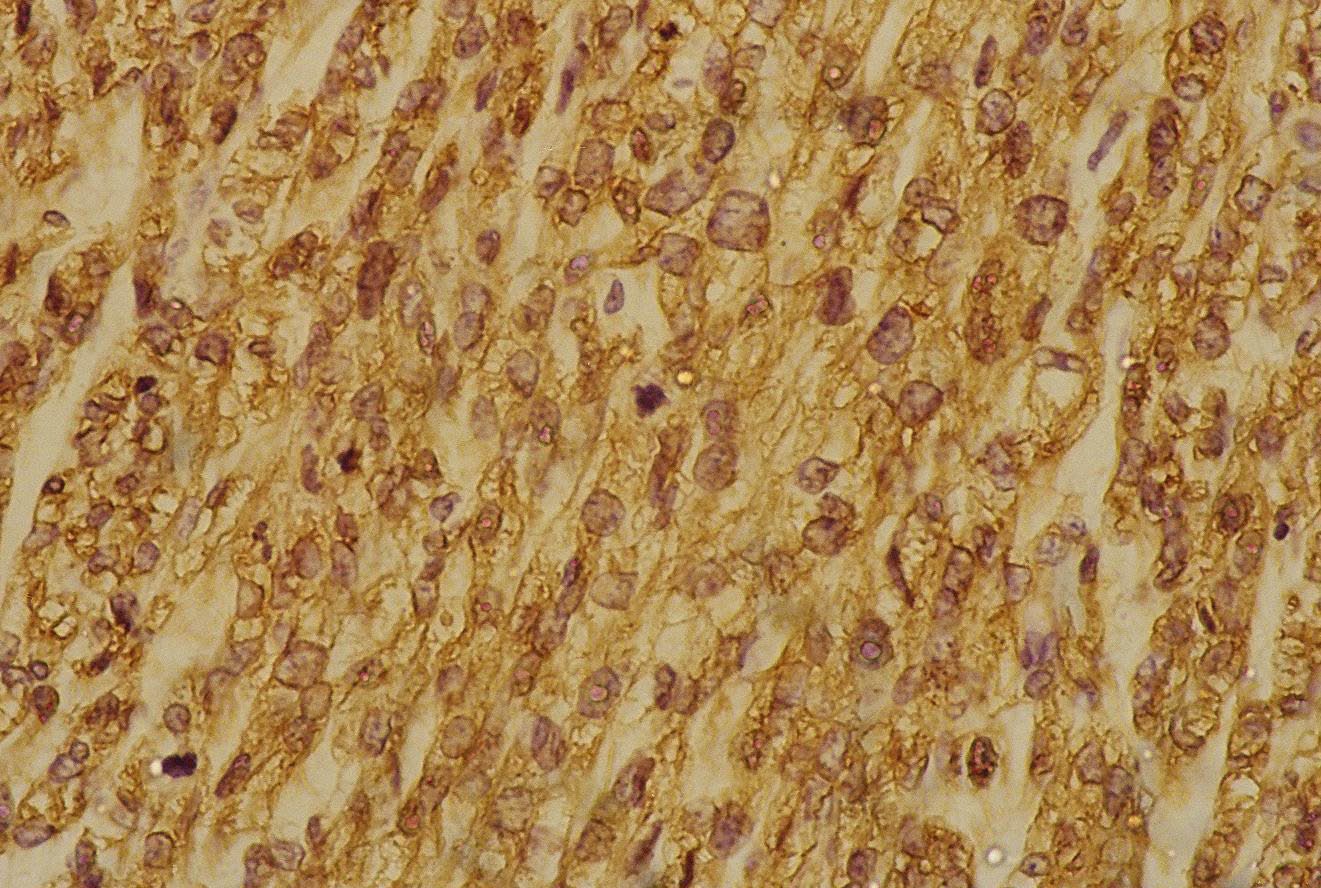

Supplement: Additional File 14 — High resolution image of figure 3f [file 1746-1596-1-13-S14.jpeg]

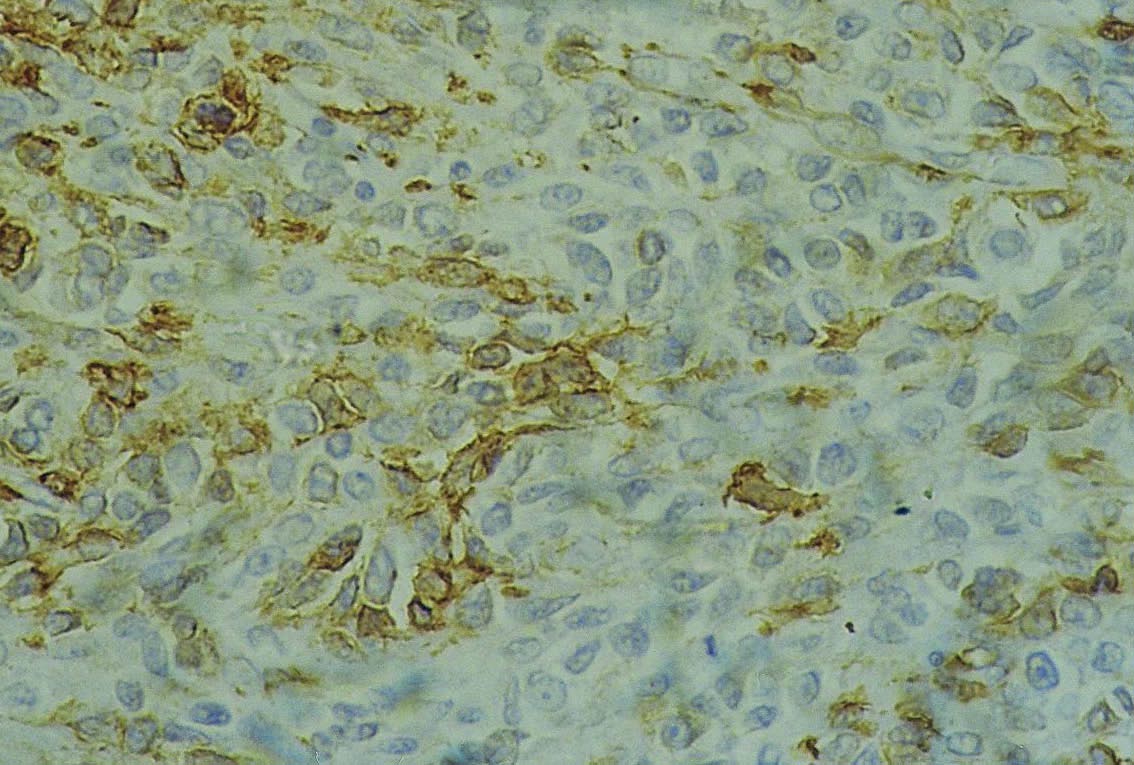

Supplement: Additional File 15 — High resolution image of figure 3g [file 1746-1596-1-13-S15.jpeg]

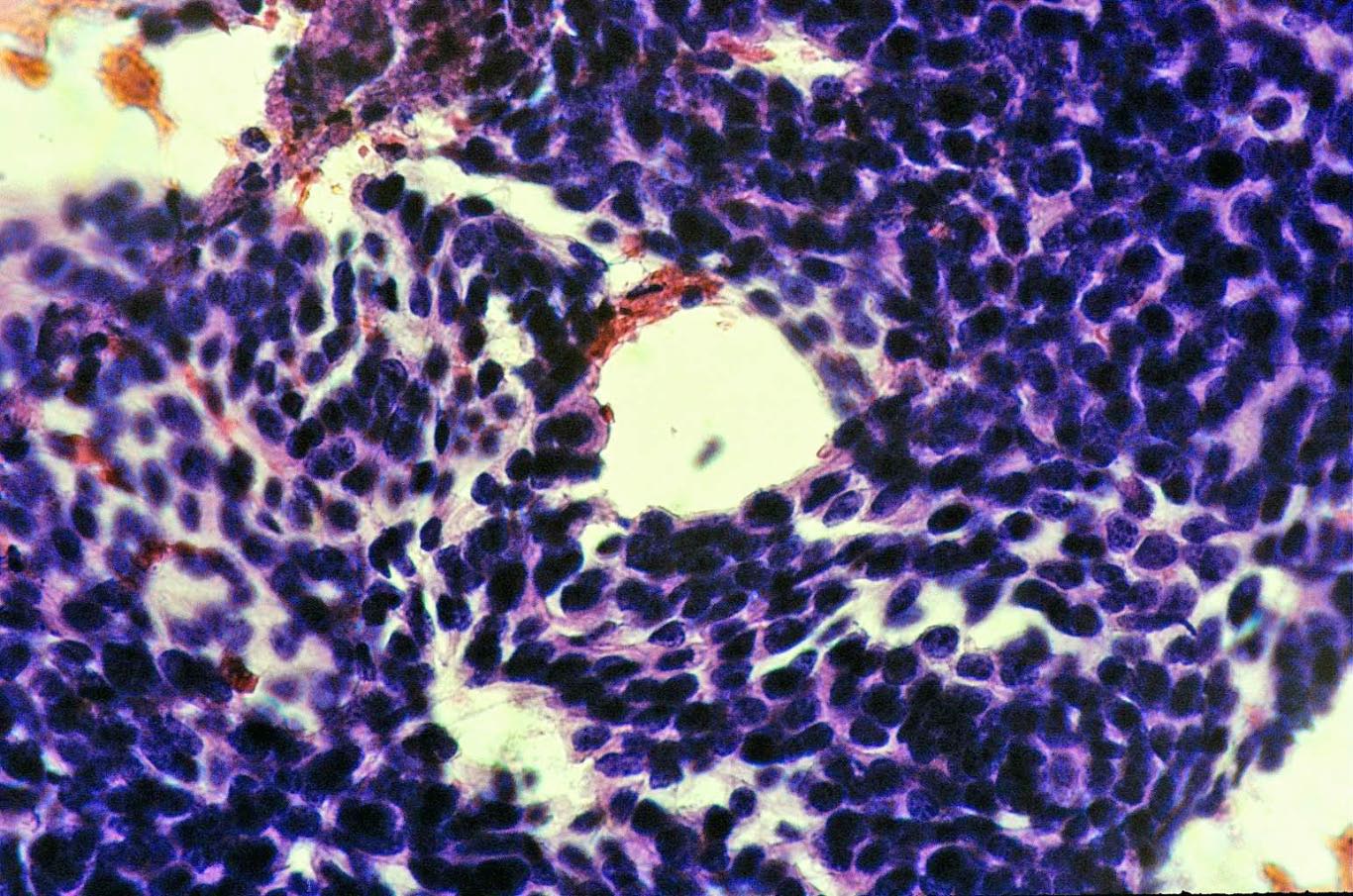

Supplement: Additional File 16 — High resolution image of figure 4a [file 1746-1596-1-13-S16.jpeg]

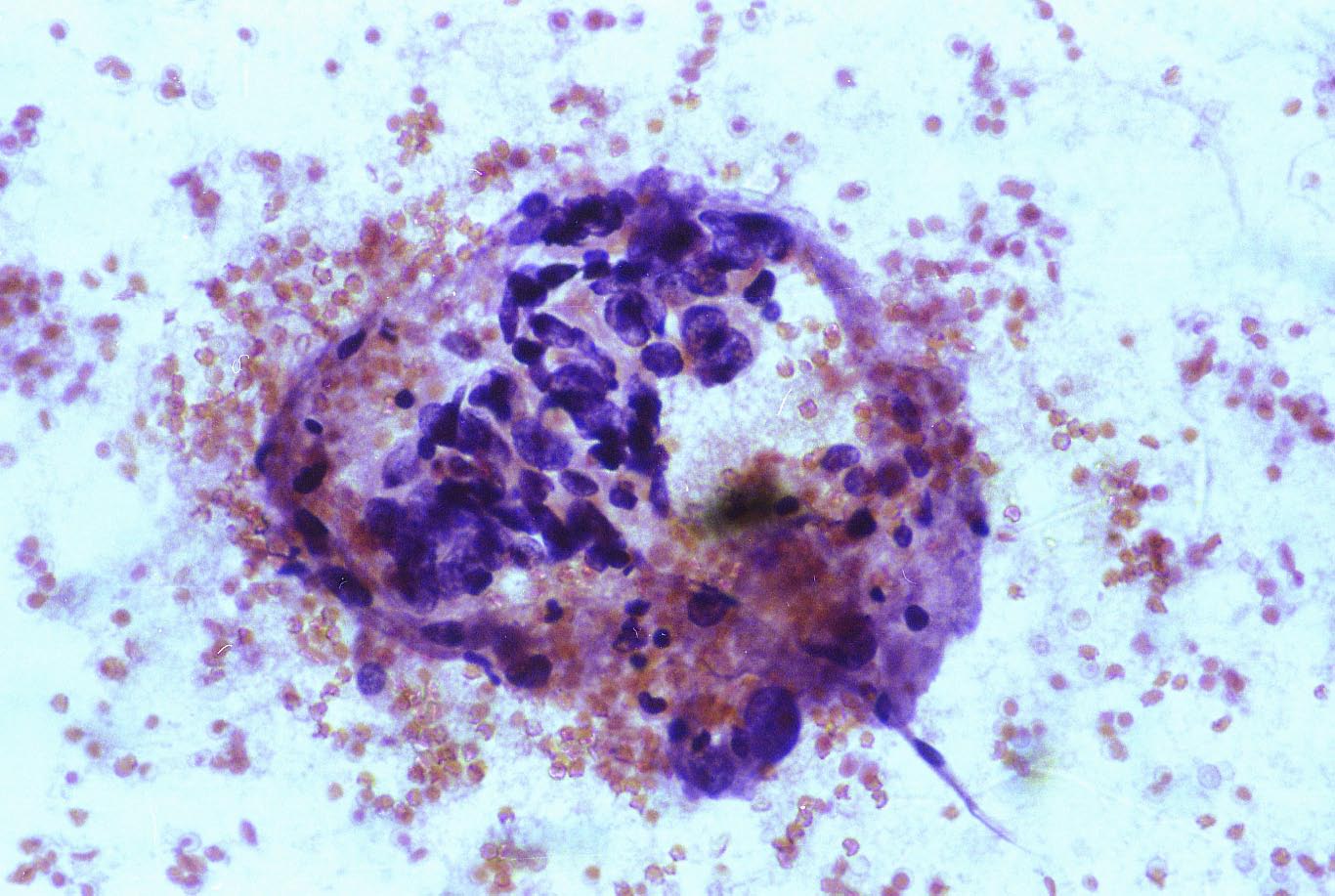

Supplement: Additional File 17 — High resolution image of figure 4b [file 1746-1596-1-13-S17.jpeg]

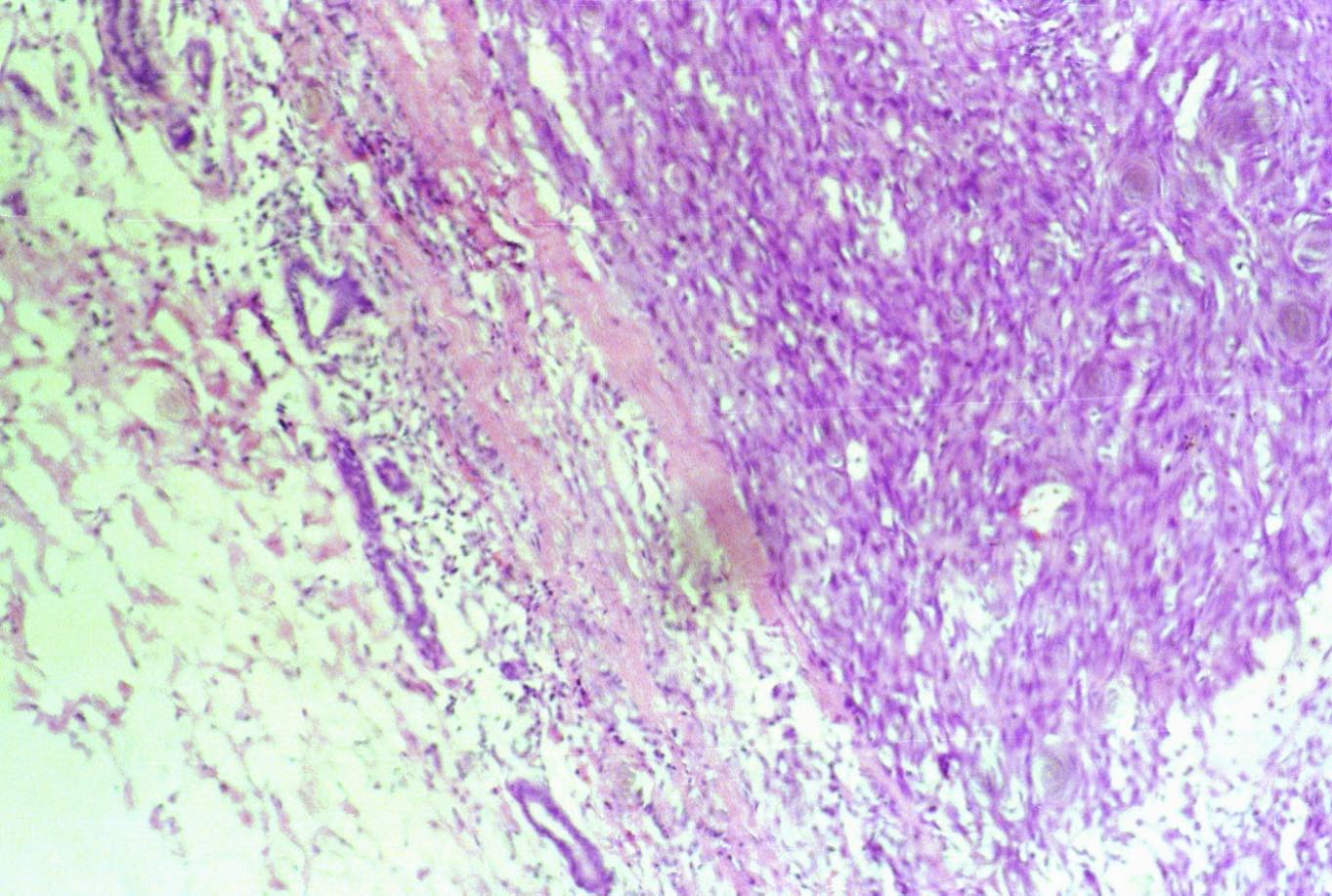

Supplement: Additional File 18 — High resolution image of figure 4c [file 1746-1596-1-13-S18.jpeg]

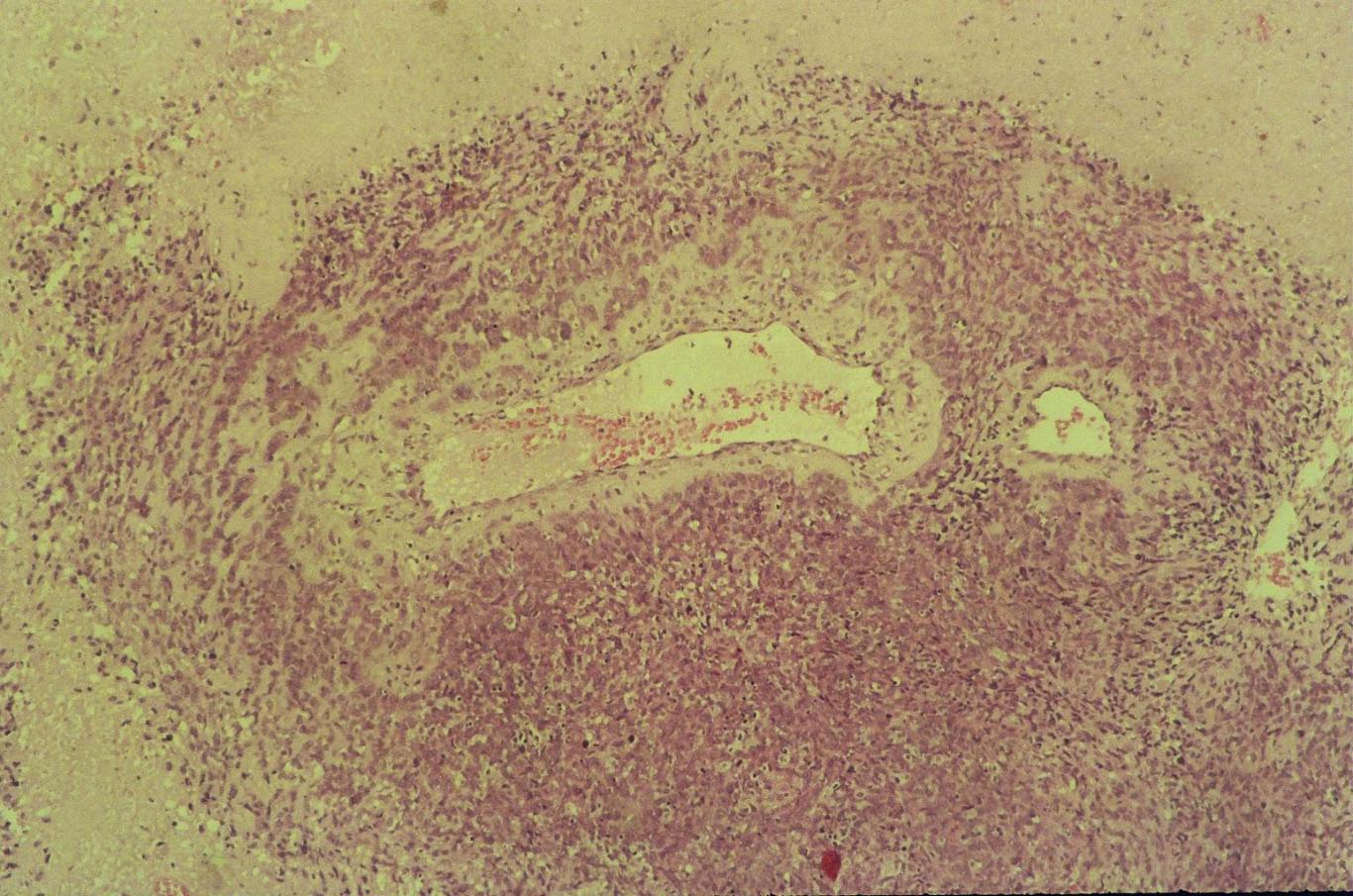

Supplement: Additional File 19 — High resolution image of figure 4d [file 1746-1596-1-13-S19.jpeg]

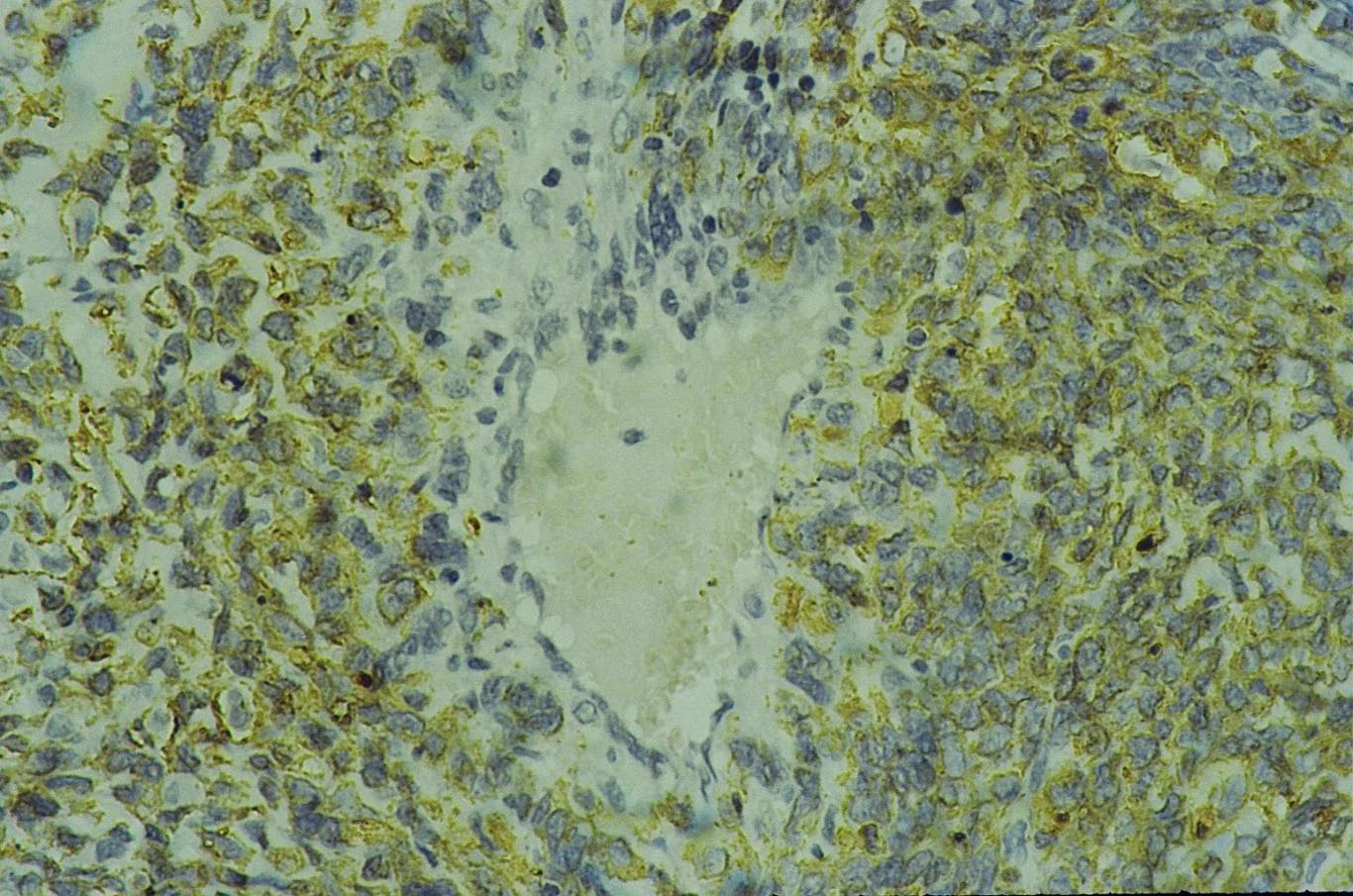

Supplement: Additional File 20 — High resolution image of figure 4e [file 1746-1596-1-13-S20.jpeg]

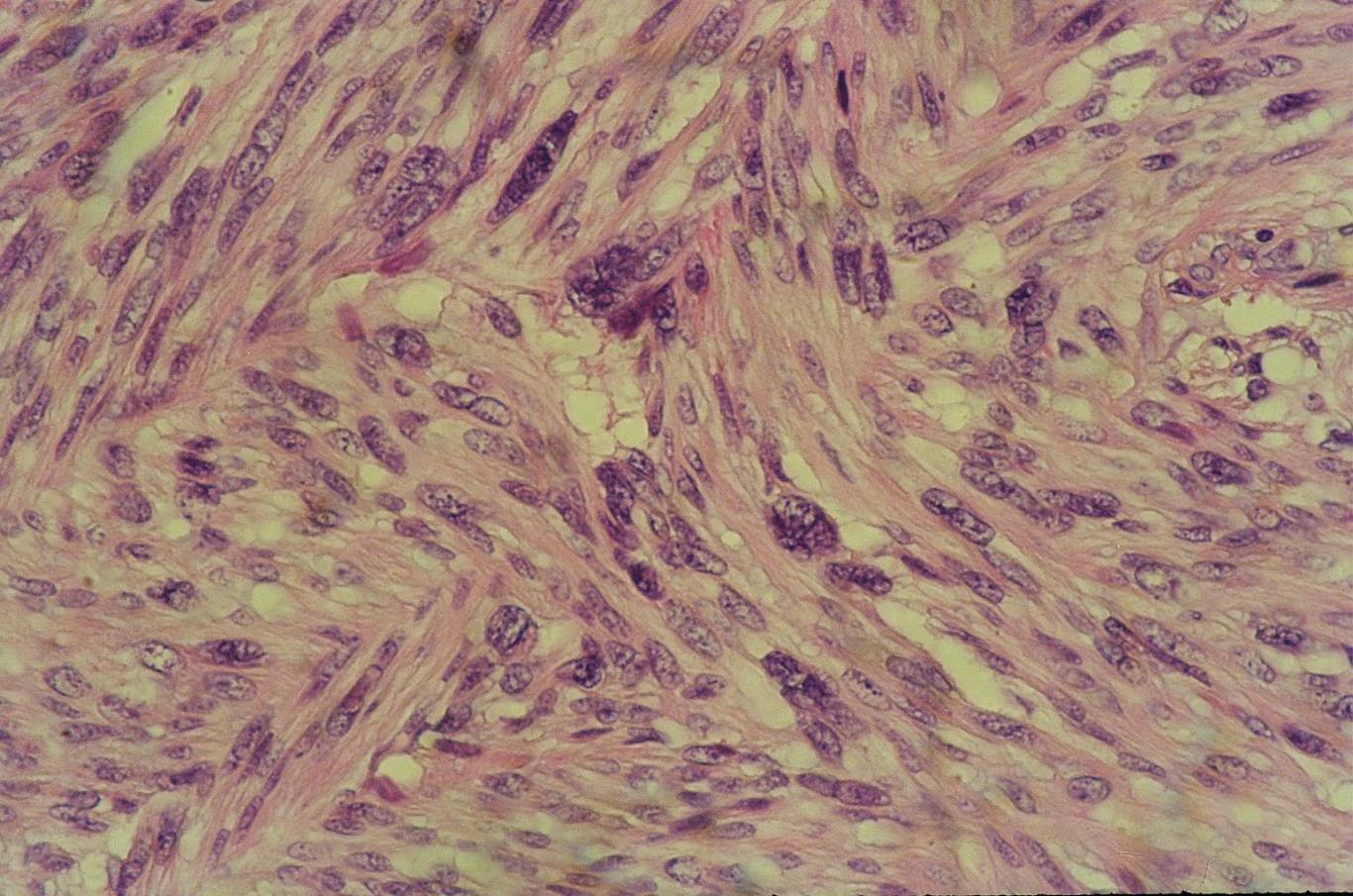

Supplement: Additional File 21 — High resolution image of figure 4f [file 1746-1596-1-13-S21.jpeg]

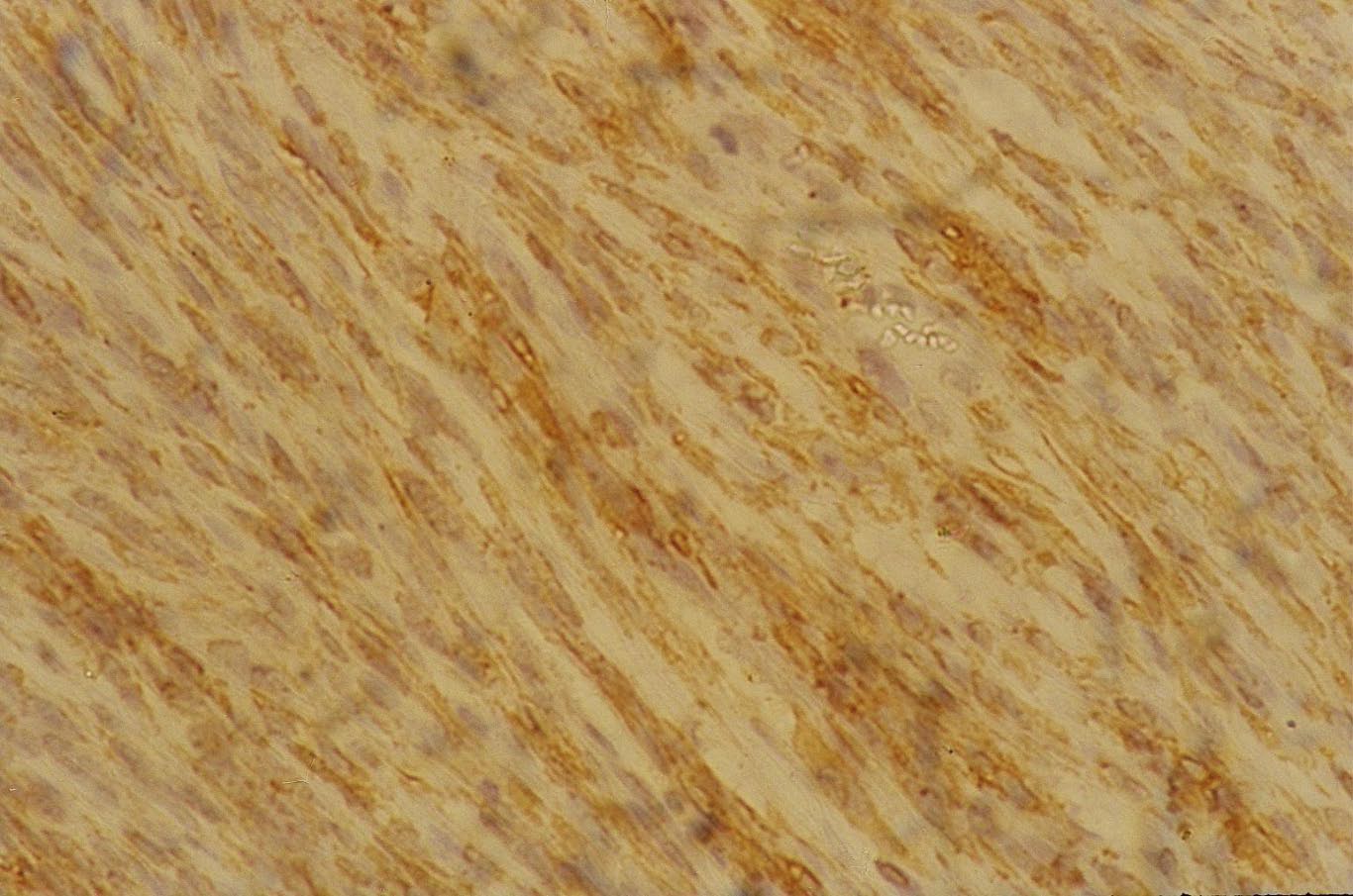

Supplement: Additional File 22 — High resolution image of figure 4g [file 1746-1596-1-13-S22.jpeg]

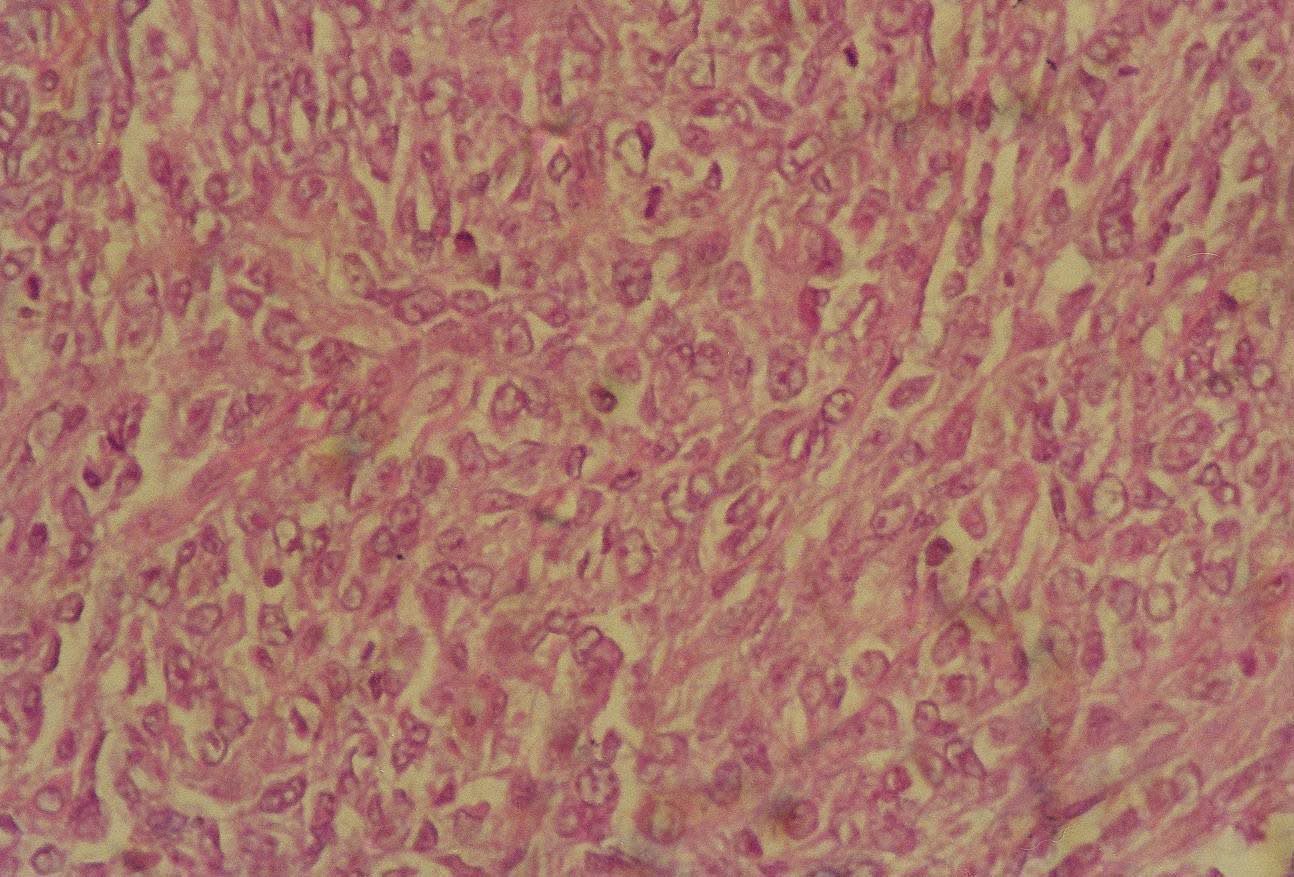

Supplement: Additional File 23 — High resolution image of figure 5a [file 1746-1596-1-13-S23.jpeg]

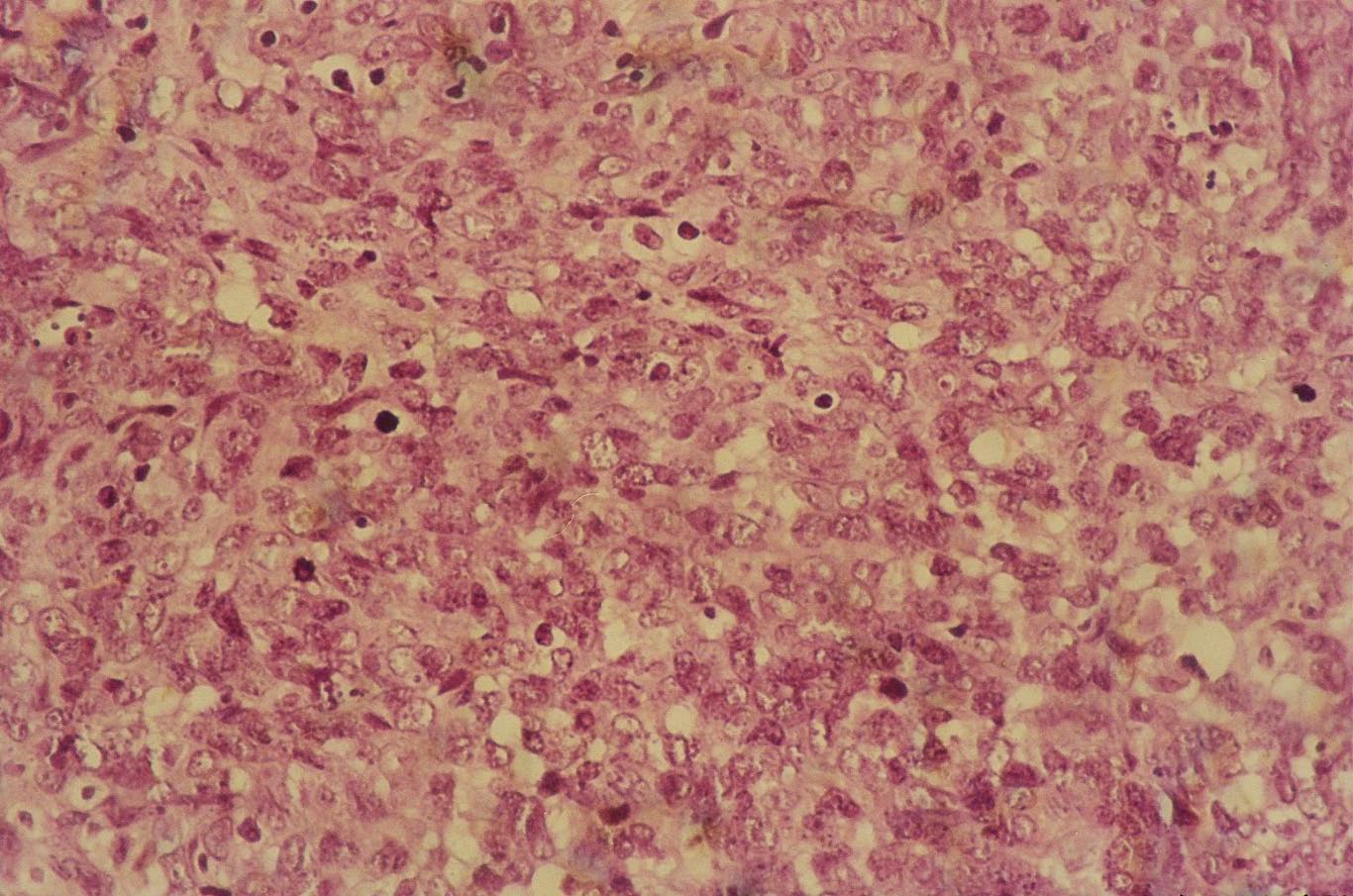

Supplement: Additional File 24 — High resolution image of figure 5b [file 1746-1596-1-13-S24.jpeg]

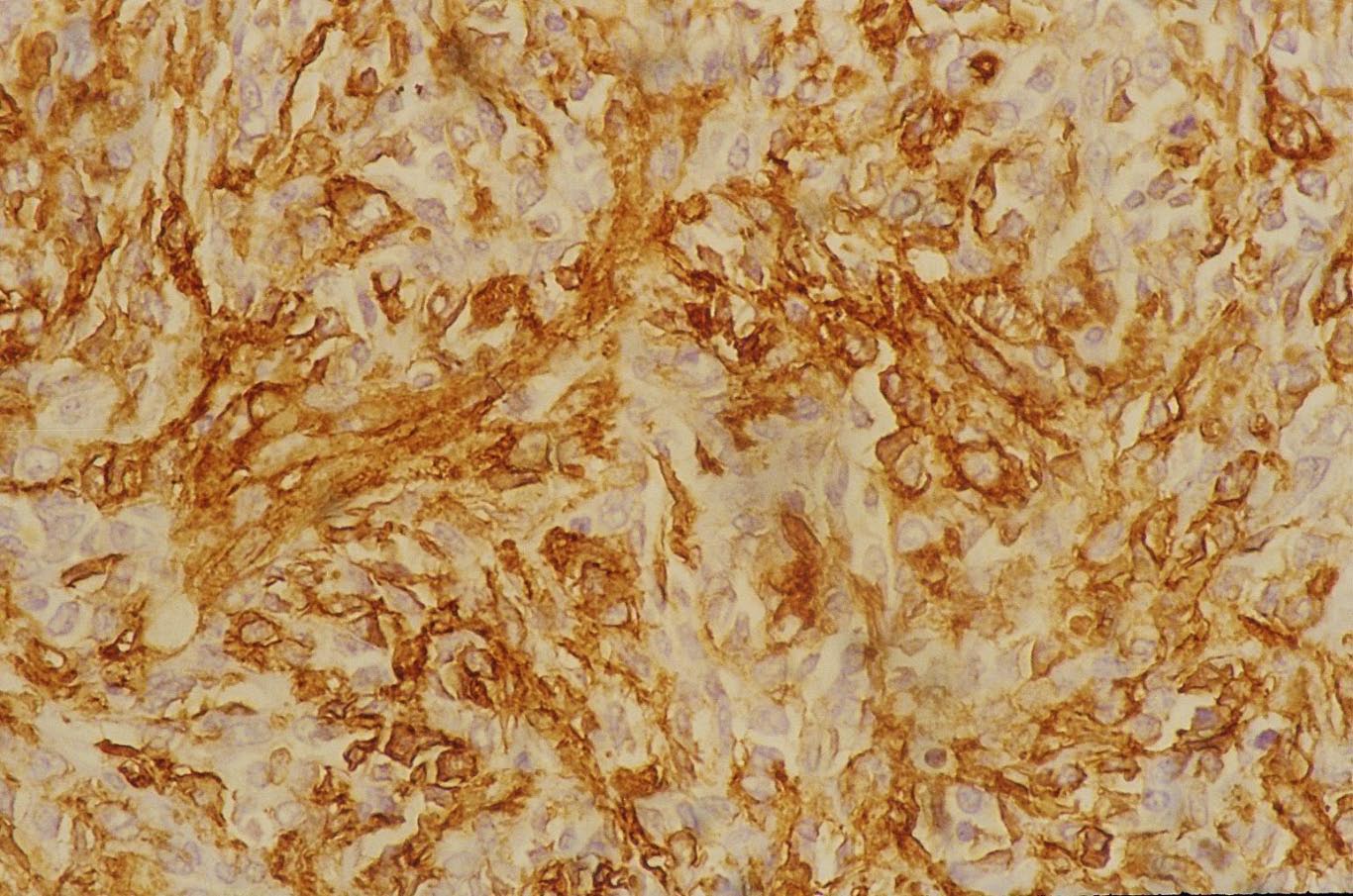

Supplement: Additional File 25 — High resolution image of figure 5c [file 1746-1596-1-13-S25.jpeg]

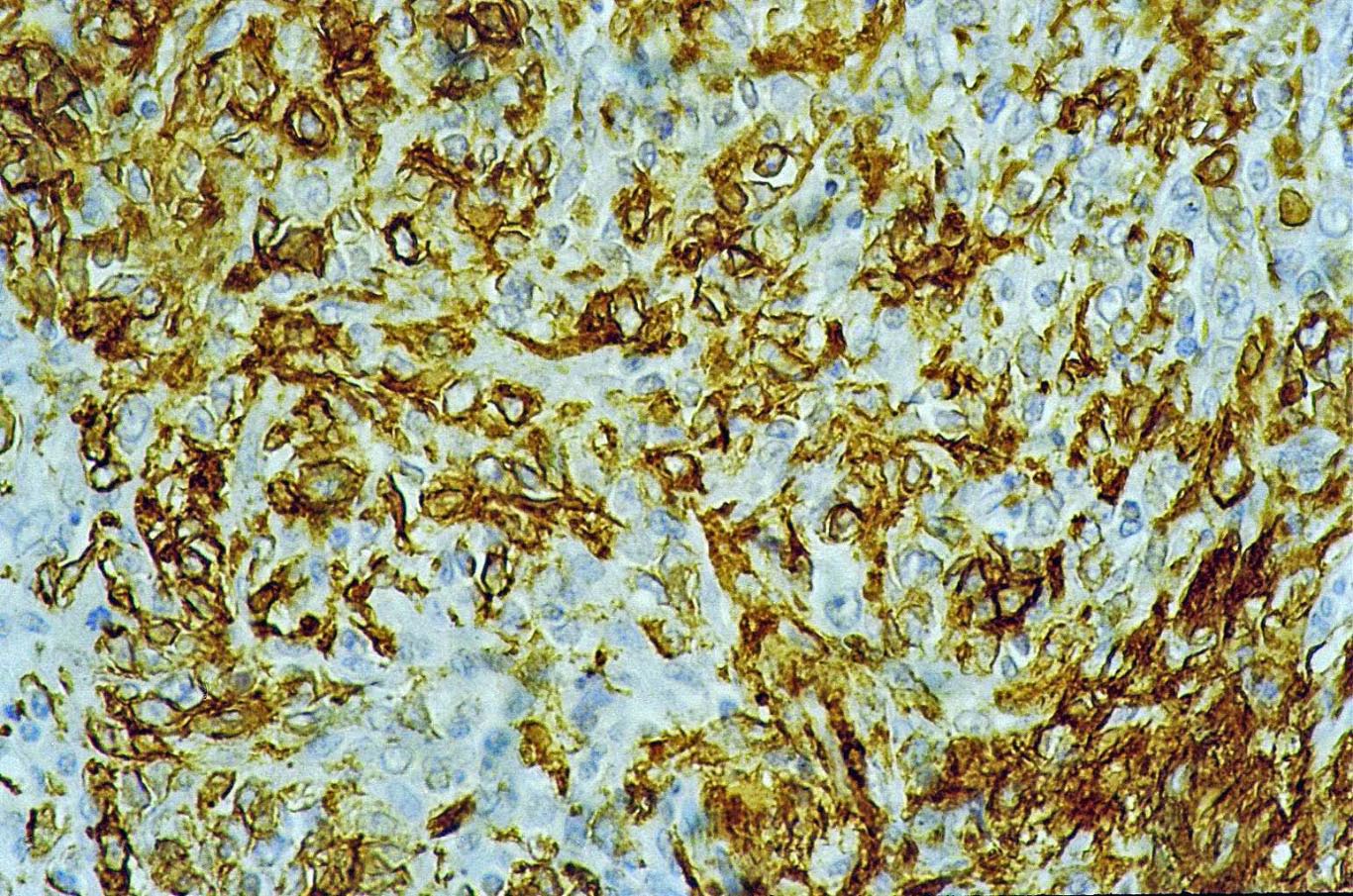

Supplement: Additional File 26 — High resolution image of figure 5d [file 1746-1596-1-13-S26.jpeg]

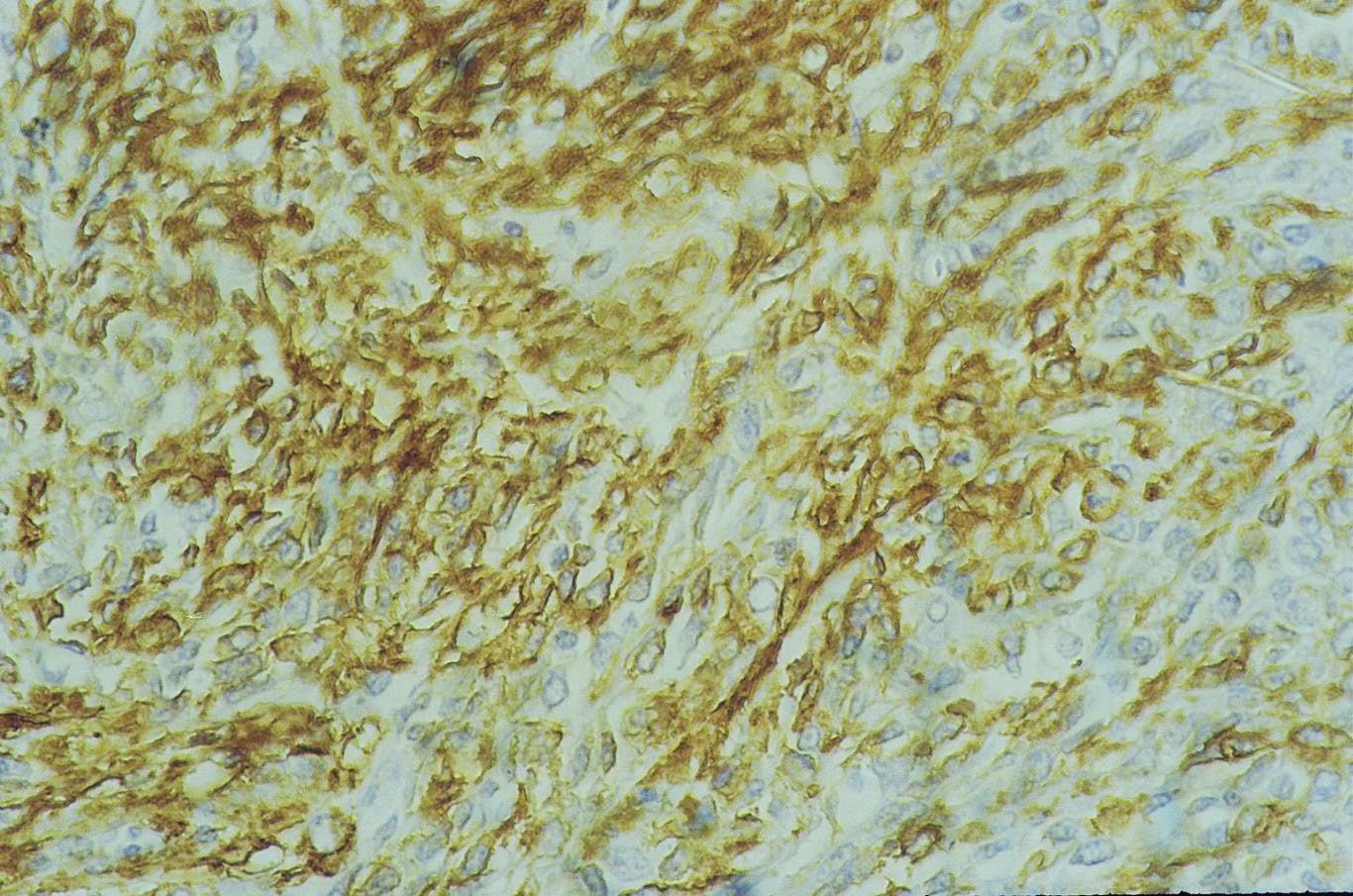

Supplement: Additional File 27 — High resolution image of figure 5e [file 1746-1596-1-13-S27.jpeg]

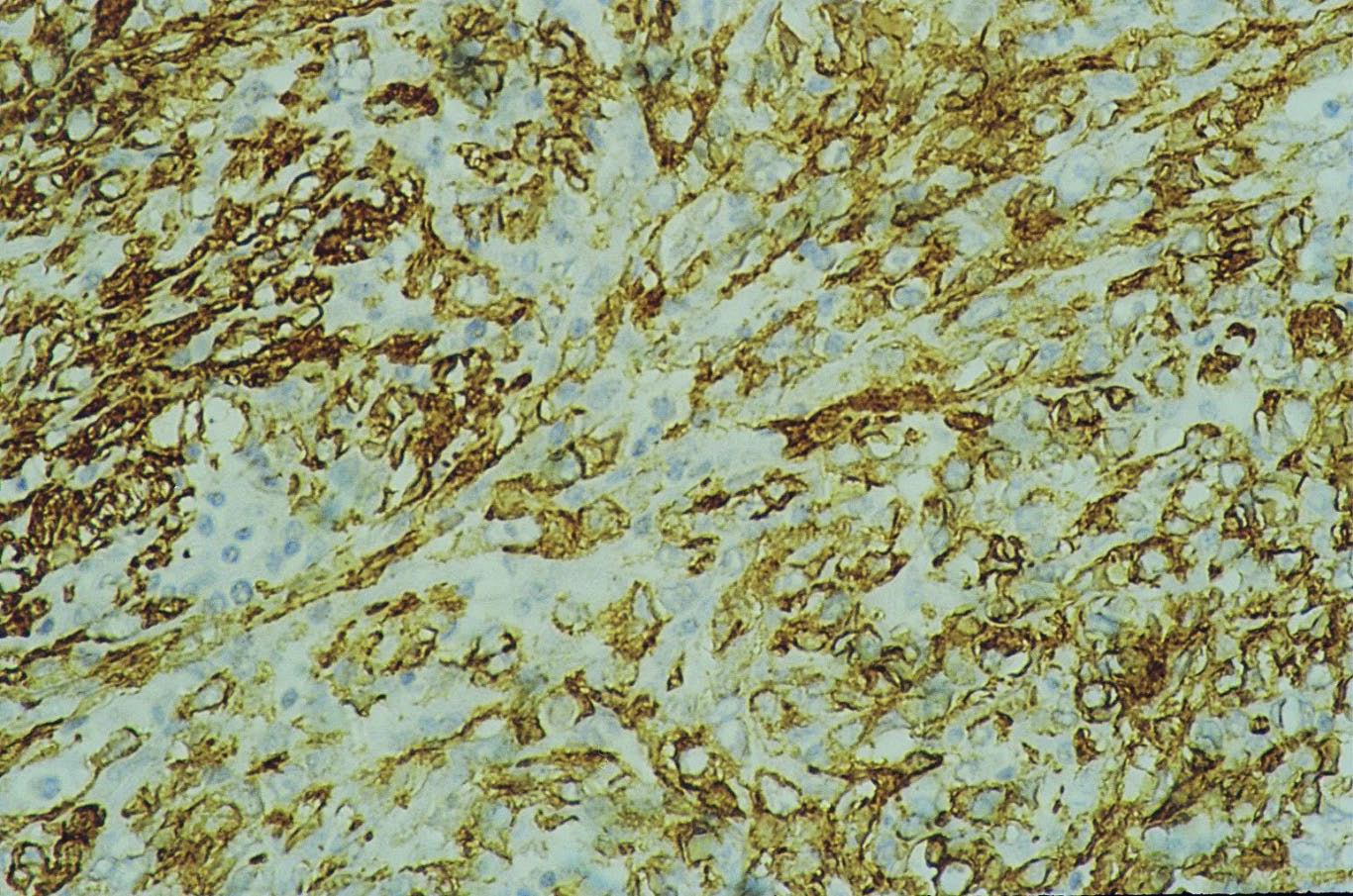

Supplement: Additional File 28 — High resolution image of figure 5f [file 1746-1596-1-13-S28.jpeg]
